# IKKβ and USP28 Regulate HEY1 Stability to Promote Cancer Stemness and Immune Evasion in Hepatocellular Carcinoma

**DOI:** 10.1002/advs.75843

**Published:** 2026-05-26

**Authors:** Na Shao, Lin Zhang, Gufang Shen, Yangfan Lv, Tianshu Fang, Ya Cao, Qiongyi Zhang, Feng Xu, Chungang Liu

**Affiliations:** ^1^ Department of Pathology Xinqiao Hospital Third Military Medical University Chongqing P. R. China; ^2^ Institute For Viral Hepatitis Key Department of Infectious Diseases Laboratory of Molecular Biology For Infectious Diseases (Ministry of Education) The Second Affiliated Hospital Chongqing Medical University Chongqing P. R. China; ^3^ Institute of Molecular and Cell Biology Agency for Science Technology and Research (A*STAR) Singapore Republic of Singapore; ^4^ Hengrui‐Singapore Innovation Centre For Chronic Diseases Singapore Republic of Singapore

**Keywords:** cancer immunotherapy, HEY1, IKKβ, protein stability, USP28

## Abstract

Hepatocellular carcinoma (HCC), the most prevalent form of primary liver cancer, is driven by cancer stem cells (CSCs) and an immunosuppressive tumor microenvironment, which may underlie the limited efficacy of immune checkpoint blockade therapy. Here, we report that HEY1 plays a crucial role in sustaining HCC stemness and undergoes polyubiquitylation during liver CSC differentiation. Mechanistically, USP28 interacts with HEY1 and deubiquitinates its lysine 87 residue, thereby stabilizing HEY1 and enhancing the stem‐like properties of liver cancer cells. Moreover, IKKβ phosphorylates HEY1 at serine 40, facilitating its interaction with USP28. Loss of USP28 reduces PD‐L1 expression, increases effector cytokine production, and suppresses tumor growth in mice. Notably, combining a USP28 inhibitor with anti‐PD‐1 immunotherapy results in enhanced tumor regression and significantly prolonged overall survival in mouse tumor models. Collectively, these findings identify USP28 as a potential biomarker for stratifying patients likely to benefit from anti‐PD‐1/PD‐L1 therapies in HCC. Furthermore, we uncover a previously unrecognized IKKβ‐USP28‐HEY1 signaling axis that governs HEY1 stability and cancer stemness, offering new opportunities for synergistic therapeutic strategies in HCC.

## Graphical abstract

The uncovered IKKβ‐USP28‐HEY1 axis fuels cancer stemness and immune evasion in hepatocellular carcinoma. USP28 deubiquitinates HEY1 upon IKKβ‐mediated phosphorylation, conferring PD‐1/PD‐L1 blockade resistance. Pharmacological inhibition of USP28 sensitizes resistant tumors to anti‐PD‐1 immunotherapy, revealing a promising therapeutic strategy.

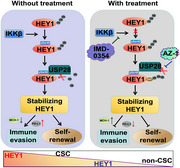

## 1 Introduction

Hepatocellular carcinoma (HCC), the most prevalent subtype of liver cancer, is characterized by poor clinical outcomes and low 5‐year survival rates [1]. The development of HCC can be driven by a small, heterogeneous population of tumor‐derived cancer stem cells (CSCs) that exhibit long‐term clonal repopulation, self‐renewal, and differentiation capacities [2, 3, 4, 5, 6]. As the cellular origin of tumor growth, CSCs must acquire intrinsic mechanisms to evade immune surveillance during tumor progression. Therefore, there is an urgent need to identify novel therapeutic targets and develop effective treatment modalities for HCC.

Tumor cells are inherently immunogenic, and the immune system plays a critical role in preventing nascent malignant cells from progressing into overt tumors. According to the CSC model, achieving complete tumor regression requires the specific targeting of CSCs [7]. Among signaling pathways governing cell fate, the Notch1‐4 cascade plays a pivotal role in determining lineage commitment, and elucidating its regulatory mechanisms is critical for controlling CSC identity, particularly in mediating transitions between distinct cellular states. As a key downstream effector of Notch signaling, hairy/enhancer‐of‐split related with YRPW motif 1 (HEY1) is an essential regulator of cell fate decisions during development [8]. Notably, aberrant HEY1 expression has been implicated in maintaining CSC self‐renewal and function across multiple human cancers, including HCC [9, 10, 11, 12]. HEY1 expression and activity are tightly regulated at both transcriptional and post‐translational levels. It undergoes various post‐translational modifications (PTMs), including phosphorylation, polymorphism, and SUMOylation [13, 14, 15]. Although ubiquitination of HEY1 has been reported [16], the precise regulatory mechanisms and functional consequences of this modification remain largely unknown.

Ubiquitination, a reversible post‐translational modification, regulates protein stability and molecular interactions through the coordinated actions of E3 ubiquitin ligases and deubiquitinating enzymes (DUBs), thereby maintaining cellular proteostasis [17, 18]. DUBs play critical roles in regulating pluripotency, cellular reprogramming, and tumorigenesis, and many have emerged as promising drug targets [19, 20, 21]. In mammals, more than 100 DUBs have been identified, with the ubiquitin‐specific protease (USP) family forming the largest subfamily, comprising approximately 54 members [22]. Ubiquitin‐specific protease 28 (USP28) is one such member and has been implicated in various physiological processes, including cell proliferation, apoptosis, self‐renewal, and differentiation [23]. Increasing evidence supports USP28's oncogenic potential, as its enzymatic activity enhances CSC self‐renewal and promotes therapy resistance [24, 25, 26]. Furthermore, USP28 has been identified as an immunomodulatory DUB, with Usp28‐deficient mice displaying a marked expansion of total T‐cell populations, particularly CD8^+^ T cells [27]. These findings highlight USP28 as a promising therapeutic target in human malignancies. Despite these findings, the role of USP28 in regulating immunotherapy responses in HCC remains unclear.

In this study, we demonstrate that HEY1 is essential for maintaining CSC self‐renewal and tumorigenicity in HCC, and that its protein levels decrease during CSC differentiation through the ubiquitin‐proteasome system. We identify USP28 as a bona fide deubiquitinase that directly stabilizes HEY1. Mechanistically, the nuclear factor kappa‐B kinase subunit beta (IKKβ) phosphorylates HEY1 at serine 40 (Ser40), thereby facilitating its interaction with USP28 and preventing ubiquitination‐mediated degradation. Moreover, genetic ablation or pharmacological inhibition of USP28 reduces PD‐L1 expression, enhances effector T‐cell activation, and significantly improves the efficacy of immunotherapy. Together, our findings uncover a molecular mechanism by which the IKKβ‐USP28 axis regulates HEY1 stability and identify a potential combinatorial therapeutic strategy to enhance anti‐tumor immune responses in HCC.

## 2 Results

### 2.1 HEY1 Promotes Self‐Renewal in Liver CSCs and Correlates With Poor Prognosis in HCC Patients

**FIGURE 1:**
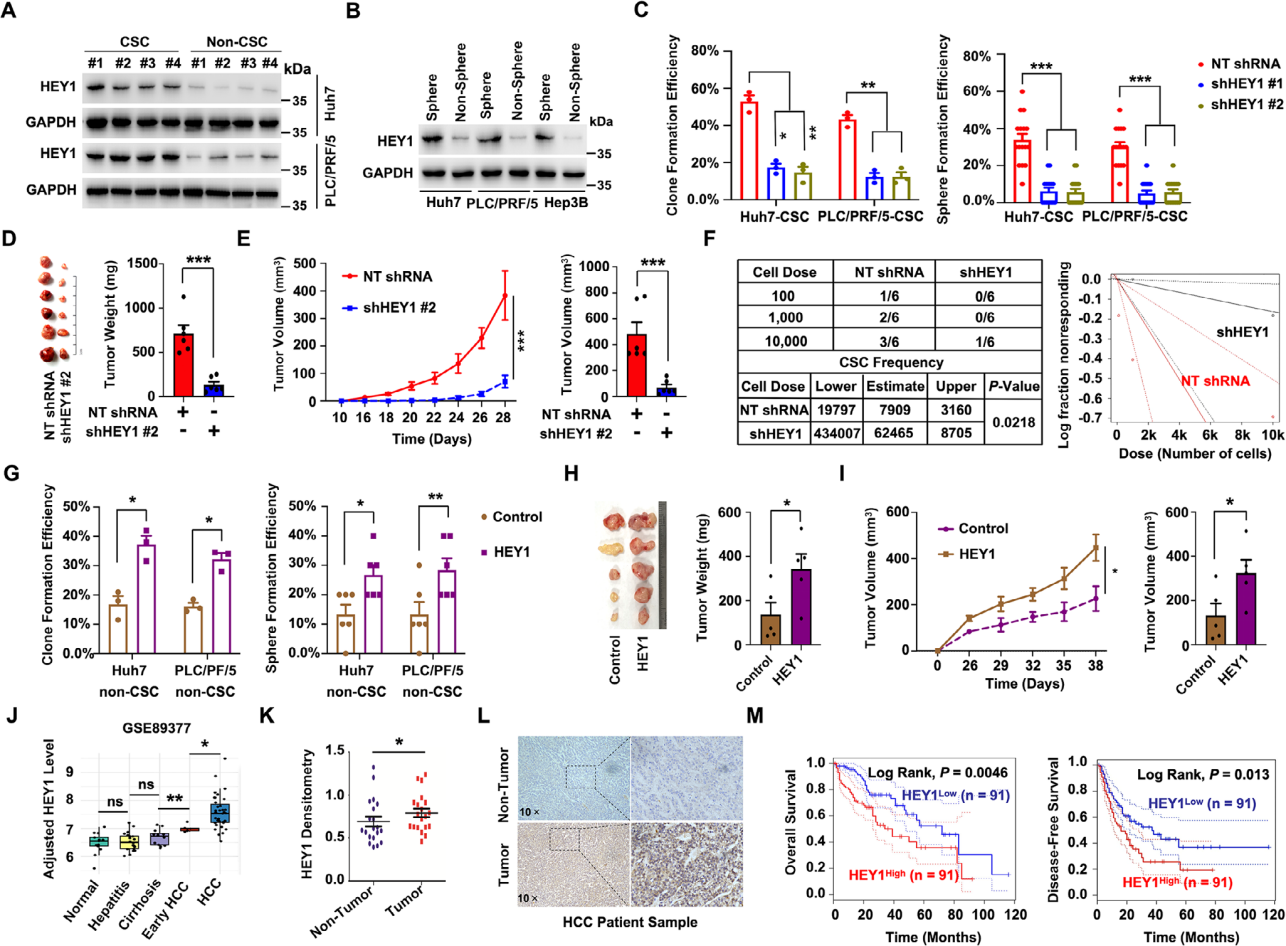
HEY1 is highly expression in liver CSCs and its positively regulates self‐renewal of liver CSCs. (A) Analysis of HEY1 expression by immunoblotting (IB) in both liver cancer stem cell (CSC) and non‐CSC tumors derived from PLC/PRF/5 and Huh7 cell lines. (B) IB analysis of HEY1 expression was performed in oncosphere and non‐oncosphere populations derived from various hepatocellular carcinoma (HCC) cell lines. (C) Colony and sphere formation efficiency assays of liver CSCs after depletion of HEY1. Data are presented as mean ± SEM from at least three independent experiments. ^*^ *p* < 0.05, ^**^ *p* < 0.01, ^***^ *p* < 0.001. *t*‐test. (D,E) Assessment of subcutaneous tumor formation from PLC/PRF/5 CSC cells with HEY1 knockdown by shRNA. Terminal tumor mass (D). Tumor growth over time (E). Data are presented as mean ± SEM. *n* = 6 per group. ^***^ *p* < 0.001. *t*‐test. (F) Impact of HEY1 depletion on PLC/PRF/5 CSC tumorigenicity assessed by limiting dilution assays. The frequency of CSCs was analyzed using the ELDA (http://bioinf.wehi.edu.au/software/elda). (G) Colony and sphere formation efficiency assays of liver non‐CSCs with stably expressing HEY1. Data are presented as mean ± SEM from at least three independent experiments. ^*^ *p* < 0.05, ^**^ *p* < 0.01. *t*‐test. (H,I) Evaluation of subcutaneous tumor formation by PLC/PRF/5 cells stably expressing HEY1. Terminal tumor mass (H). Tumor growth over time (I). *n* = 5 per group. ^*^ *p* < 0.05. *t*‐test. (J) HEY1 expression in liver tissues across different disease phases in the GSE89377 dataset. (K) Quantification of HEY1 protein levels by densitometry for IB in non‐tumor tissues and tumors of HCC patients. ^*^ *p* < 0.05. *t*‐test. (L) Immunohistochemical (IHC) staining of HCC samples for HEY1. Representative images are shown. (M) Kaplan‐Meier curves showing overall survival and disease‐free survival of HCC patients based on HEY1 expression.

Previous studies have suggested that HEY1 contributes to HCC progression by enhancing the self‐renewal capacity of tumor cells [9]. To further explore this observation, we examined HEY1 expression in freshly purified liver CSCs, differentiated cancer cells, and xenograft tumors, as previously described [28]. Our analyses revealed that HEY1 protein levels were markedly elevated in CSCs compared with non‐CSCs (Figure 1A and Figure S1A–C). Similar results were observed in oncosphere cells derived from HCC cell lines (Figure 1B). To determine the functional role of HEY1 in liver CSC self‐renewal, we first assessed whether knockdown (KD) of HEY1 expression would impair their stemness and tumorigenic potential. HEY1‐KD significantly reduced clonogenicity and sphere‐forming efficiency in liver CSCs (Figure 1C and Figure S1D). Moreover, in an established xenograft tumor model, HEY1‐KD CSCs required a higher number of cells and longer latency to form tumors of comparable size to those produced by control CSCs (Figure 1D,E). Limiting‐dilution analysis further confirmed that control cell populations were significantly enriched for CSCs relative to their HEY1‐KD counterparts (Figure 1F). Together, these findings demonstrate that HEY1 is highly expressed in liver CSCs and is functionally indispensable for maintaining their self‐renewal capacity.

We next investigated whether HEY1 could restore self‐renewal in non‐CSC populations. Lentiviral‐mediated ectopic expression of HEY1 in non‐CSCs (Figure S1E) significantly enhanced both sphere‐ and colony‐forming abilities (Figure 1G). Furthermore, HEY1‐overexpressing non‐CSCs displayed markedly increased tumorigenic potential compared with control non‐CSCs (Figure 1H,I). Consistent with these experimental results, HEY1 protein levels were substantially elevated in tumor tissues from HCC patients (Figure 1J–L and Figure S1F). Bioinformatic analyses of HCC patient datasets revealed significant enrichment of the HEY1 signature in advanced‐stage and higher‐grade tumors (Figure S1G). Importantly, Kaplan‐Meier survival analysis demonstrated that high HEY1 expression was strongly associated with poor overall survival and shorter disease‐free survival in HCC patients (Figure 1M and Figure S1H,I). Collectively, these data provide compelling evidence that HEY1 plays a crucial role in sustaining the stem‐like phenotype of liver CSCs and contributes to HCC progression and poor clinical outcomes.

### 2.2 HEY1 is Ubiquitylated During Liver CSC Differentiation

**FIGURE 2:**
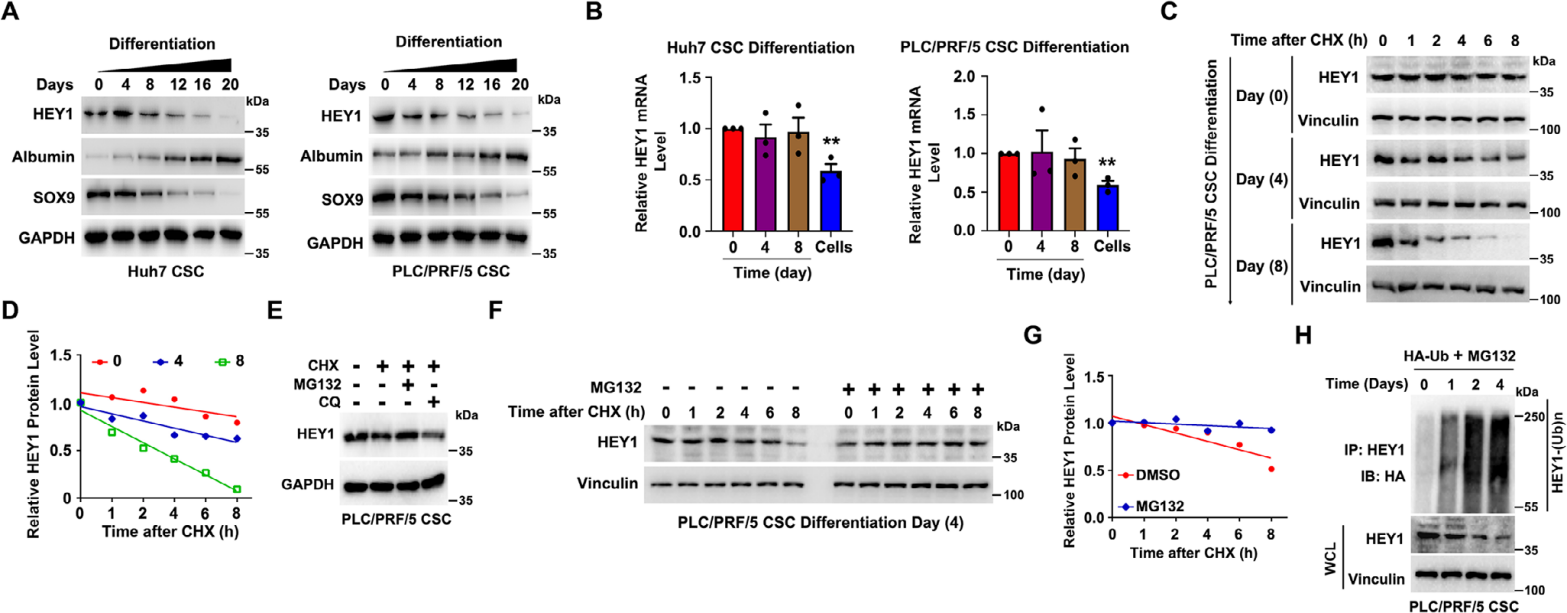
HEY1 undergoes ubiquitin‐proteasome‐mediated degradation during liver CSC differentiation. (A) IB analysis was performed using the indicated antibodies to monitor protein expression dynamics at various time points during the in vitro differentiation of liver CSCs. (B) QRT‐PCR analysis of HEY1 mRNA levels at different times during liver CSCs differentiation in vitro. (C,D) Liver CSCs were cultured in medium containing 10% FBS to induce differentiation for the indicated times. Cells were treated with cycloheximide (CHX, 100 µg/mL) and collected at the indicated times for IB (C). The HEY1 protein abundance was quantified by the ImageJ software and plotted (D). (E) IB analysis of HEY1 protein levels in PLC/PRF/5 CSC cells treated with 100 µg/mL CHX alone or in combination with 20 µm MG132 or 10 µm chloroquine (CQ) for 8 h prior to cell harvesting. (F,G) IB analysis of HEY1 protein levels in PLC/PRF/5 CSC cells following treatment with either 100 µg/mL CHX alone or in combination with 20 µm MG132, and collected at the indicated times for IB (F). The HEY1 protein abundance was quantified by the ImageJ software and plotted (G). H PLC/PRF/5 CSCs expressing HA‐tagged ubiquitin (HA‐Ub) were exposed to the proteasome inhibitor MG132 (20 µm) for 8 h prior to harvest. HEY1 protein was subsequently immunoprecipitated using a specific anti‐HEY1 antibody, and ubiquitination was detected by immunoblotting with an anti‐HA antibody.

To investigate the dynamics of HEY1 expression during liver CSC differentiation under conventional culture conditions, as previously described [29], we observed a gradual decline in HEY1 protein levels accompanied by increased expression of the hepatocellular differentiation marker albumin (Figure 2A). Notably, HEY1 mRNA levels remained largely unchanged throughout the 8‐day differentiation period (Figure 2B), suggesting post‐transcriptional regulation. To further elucidate the mechanisms governing HEY1 stability, liver CSCs were treated with cycloheximide (CHX) to block protein synthesis. The results revealed that HEY1 exhibited a markedly shortened half‐life following differentiation induction (Figure 2C,D). Conversely, treatment with the proteasome inhibitor MG132, but not the lysosomal inhibitor chloroquine (CQ), led to a pronounced accumulation of endogenous HEY1 protein (Figure 2E), primarily by prolonging its half‐life (Figure 2F,G). In addition, analysis of HEY1 ubiquitination during differentiation revealed a substantial increase in ubiquitinated HEY1 following differentiation induction (Figure 2H). Collectively, these findings demonstrate that HEY1 protein stability is tightly controlled by the ubiquitin‐proteasome system during liver CSC differentiation, underscoring the importance of ubiquitination in regulating CSC fate determination.

### 2.3 USP28 Deubiquitylates and Stabilizes HEY1

**FIGURE 3:**
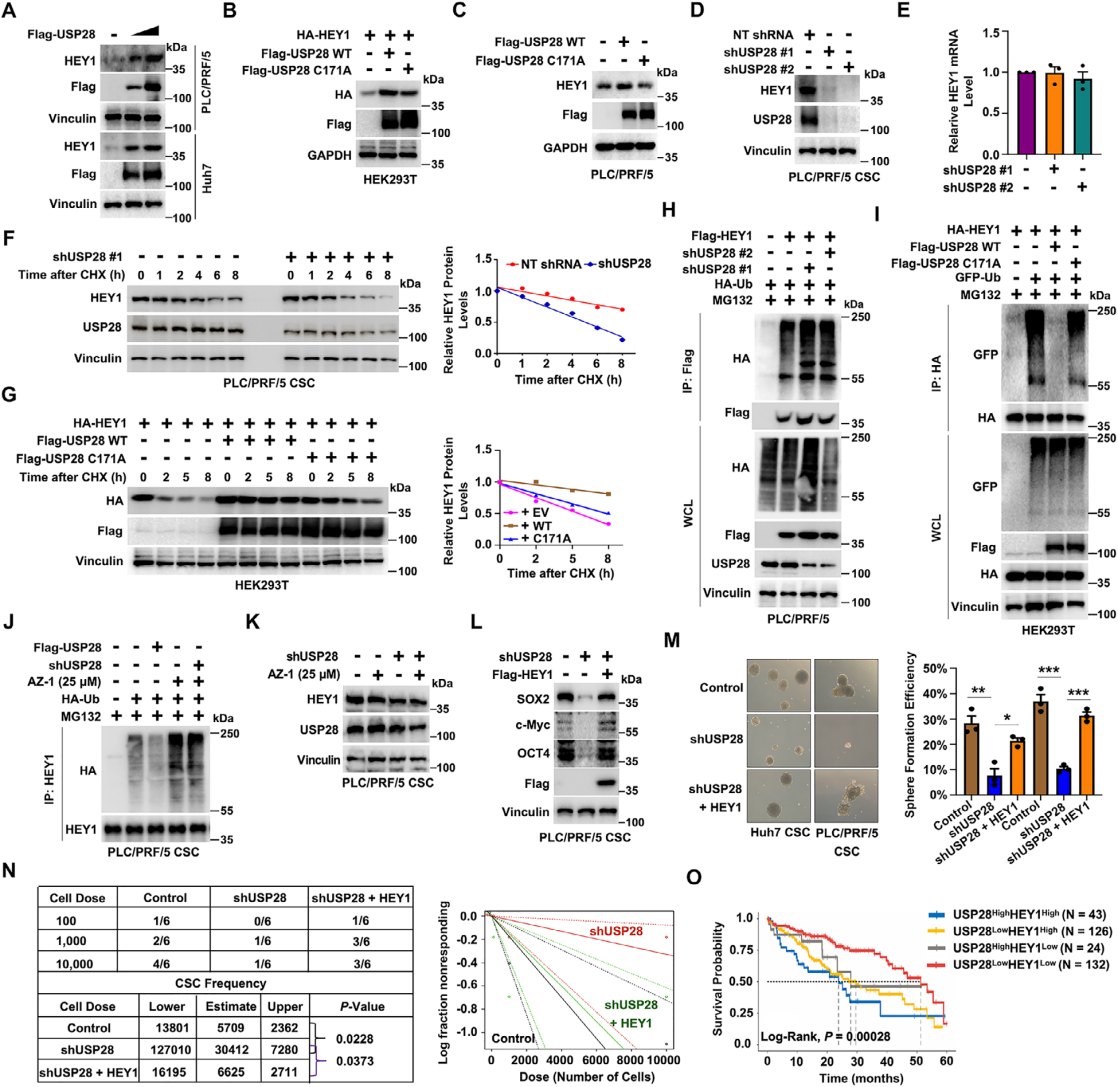
USP28 regulates HEY1 protein stability to promote oncogenicity. (A) HEY1 protein levels were assessed by immunoblotting in PLC/PRF/5 and Huh7 cells expressing increasing amounts of Flag‐USP28 (250 or 500 ng plasmid DNA). Cell lysates were prepared 48 h after transfection with the indicated constructs. (B,C) USP28 stabilizes HEY1 in an activity‐dependent manner. IB analysis of WCL from HEK293T and PLC/PRF/5 cells transfected with the indicated plasmids. (D,E) IB (D) and qRT‐PCR (E) analysis of HEY1 from PLC/PRF/5 CSC cells with USP28 knockdown (KD). *n* = 3. (F) IB analysis of WCL from PLC/PRF/5 CSC cells with the depletion of USP28 and treated with 100 µg/mL CHX for indicated time points before harvesting (Left Panel). The HEY1 protein abundance was quantified by the ImageJ software and plotted (Right Panel). (G) IB analysis of HEY1 protein stability in HEK293T cells expressing indicated constructs. Cells were treated with CHX (100 µg/mL) 36 h post‐transfection and lysed at indicated time points (Left Panel). HEY1 band intensity was quantified using ImageJ and plotted over time (Right Panel). (H) To assess HEY1 ubiquitination, USP28‐depleted PLC/PRF/5 cells were treated with MG132 (20 µm, 8 h). HEY1 was immunoprecipitated with anti‐Flag antibody, and ubiquitination was detected by anti‐HA immunoblotting, targeting the HA tag on ubiquitin. (I) Effects of USP28 catalytic activity on HEY1 polyubiquitination. IB analysis of WCL and IP products derived from HEK293T cells transfected with indicated constructs. 36 h post transfection, cells were treated with 20 µm MG132 for 8 h before harvesting. (J) Effects of AZ‐1 on USP28‐mediated HEY1 polyubiquitination. PLC/PRF/5 CSC cells expressing indicated plasmids were treated with 25 µm AZ‐1. The ubiquitinated HEY1 was immunoprecipitated anti‐HEY1 and analyzed by IB. (K) IB analysis of HEY1 protein levels in PLC/PRF/5 CSCs treated with 25 µm AZ‐1, or with USP28 depletion, or with both together. (L) IB analysis of HEY1 protein levels in PLC/PRF/5 CSCs treated with 25 µm AZ‐1, with or without transfection of the indicated USP28 plasmids. (M) IB analysis of indicted proteins obtained from PLC/PRF/5 CSC cells with USP28 depletion or stable expression of HEY1 with endogenous USP28 knockdown. (N) Sphere formation assays were conducted on Huh7 or PLC/PRF/5 cells with USP28 depletion, or stable expression of HEY1 with endogenous USP28 knockdown. ^*^ *p* < 0.01, ^**^ *p* < 0.01,^***^ *p* < 0.001. *t*‐test. (O) Effects of USP28 depletion or stable expression of HEY1 with endogenous USP28‐KD on the tumor‐forming frequency of PLC/PRF/5 CSCs for extreme limiting dilution analysis. (P) Kaplan‐Meier survival analysis indicated that expression levels of both USP28 and HEY1 were significantly correlated with overall survival outcomes in patients with HCC.

Ubiquitination and deubiquitination, catalyzed by E3 ubiquitin ligases and deubiquitinases, respectively, constitute a reversible post‐translational mechanism that regulates protein homeostasis and diverse cellular functions [17, 18]. To identify potential DUBs that stabilize HEY1, we screened a previously described DUB cDNA library [19]. Among the DUBs tested, only USP28 markedly increased HEY1 protein abundance (Figure S2A), mimicking the effect of the MG132. In contrast, USP25, the closest homolog of USP28, failed to stabilize HEY1 (Figure S2B,C). Ectopic expression of USP28 elevated HEY1 protein levels in a dose‐dependent manner (Figure 3A and Figure S2D), whereas expression of the catalytically inactive mutant USP28 C171A (Cys171→Ala) abolished this effect (Figure 3B,C and Figure S2E). Conversely, USP28 depletion reduced HEY1 protein levels, an effect that was rescued by MG132 treatment (Figure 3D and Figure S2F,G). Importantly, HEY1 mRNA levels remained unchanged (Figure 3E), indicating post‐transcriptional regulation. The half‐life of HEY1 was significantly shortened in USP28‐depleted cells and prolonged upon USP28 overexpression (Figure 3F,G and Figure S2H,I), which corresponded to increased or decreased HEY1 polyubiquitylation, respectively (Figure 3H,I and Figure S2J). Consistently, the USP28 C171A mutant failed to extend HEY1 stability (Figure 3G). In vivo deubiquitination assays further confirmed that HEY1 polyubiquitylation was efficiently removed by wild‐type USP28 but not by the catalytic mutant (Figure 3I and Figure S2K).

Moreover, treatment with the USP28 inhibitor AZ‐1 led to robust HEY1 degradation (Figure S2L–N). Combined USP28 depletion and AZ‐1 treatment did not further increase HEY1 polyubiquitylation or reduce its protein levels compared with either intervention alone (Figure 3J,K), suggesting a shared mechanism of action. Consistently, ectopic expression of wild‐type USP28, but not the C171A mutant, partially rescued HEY1 protein levels in the presence of AZ‐1 (Figure 3L). Collectively, these data demonstrate that USP28 directly deubiquitylates and stabilizes HEY1 by preventing its proteasomal degradation.

We next sought to elucidate the biological significance of USP28‐mediated HEY1 stabilization. Given that HEY1 is a key regulator of CSC self‐renewal and tumorigenic potential (Figure 1), we examined whether USP28 plays a similar role. USP28 protein levels were markedly elevated in CSCs compared with non‐CSC populations (Figure S3A,B). Consistently, expression of multiple CSC‐related genes, including SOX2, OCT4, and c‐MYC, was downregulated upon USP28 depletion and upregulated upon USP28 overexpression (Figure S3C–F). Treatment with the USP28 inhibitor AZ‐1 also decreased CSC gene expression in a dose‐ and time‐dependent manner (Figure S3G–J). In agreement, either genetic ablation or pharmacological inhibition of USP28 significantly reduced sphere formation and clonogenic growth (Figure S3K,L). Elevated USP28 expression correlated with worse overall and disease‐free survival in HCC patients (Figure S3M). Importantly, stable expression of HEY1 in USP28‐depleted cells restored CSC‐related gene expression, sphere formation, and tumorigenic potential (Figure 3M–O). Bioinformatic analyses further revealed significant enrichment of the HEY1 transcriptional signature in tumors with high USP28 expression (Figure S3N), and co‐expression of USP28 and HEY1 was associated with poor overall survival (Figure 3P). Together, these findings establish a mechanistic model in which USP28 promotes HCC progression by deubiquitylating and stabilizing HEY1, thereby sustaining CSC‐like properties and enhancing tumor aggressiveness.

### 2.4 USP28 Deubiquitinates HEY1 at Lysine 87 to Confer Oncogenic Activity

**FIGURE 4:**
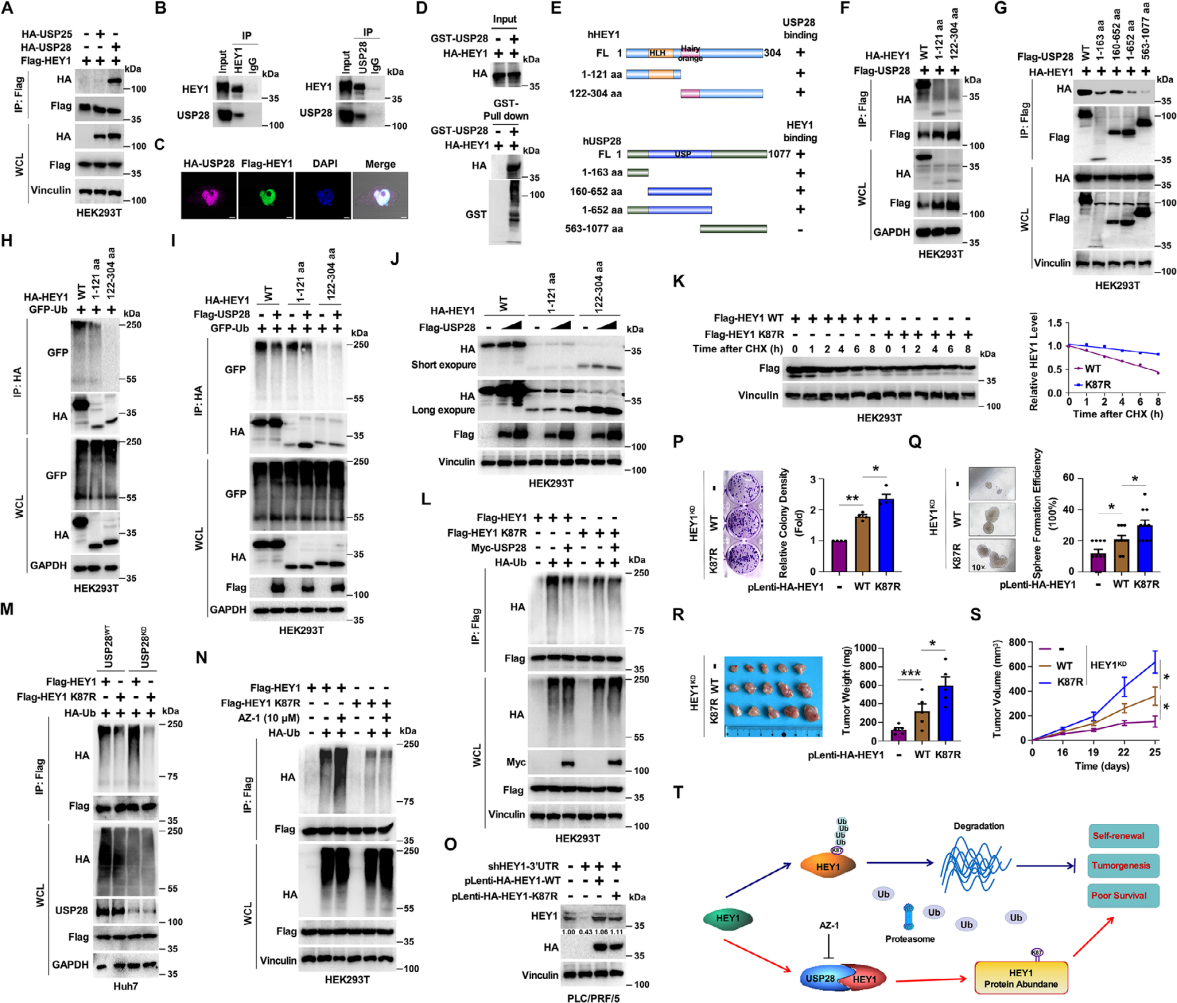
USP28 deubiquitinates HEY1 at lysine 87 to promote oncogenicity. (A) Identification of USP28 as a specific deubiquitinase (DUB) interacting with HEY1. IB analysis of WCL and Flag immunoprecipitate from HEK293T cells transfected with Flag‐HEY1 and HA‐tagged USP25 or USP28. (B) Endogenous interaction between USP28 and HEY1 was confirmed by co‐IP in PLC/PRF/5 cells. Lysates were immunoprecipitated with antibodies against either USP28 or HEY1 and analyzed by immunoblotting. Input is 5% of the total lysates used in IP. (C) HA‐USP28 was co‐expressed with Flag‐HEY1 in PLC/PRF/5 cells. The subcellular localization of USP28 (Magenta) and HEY1 (Green) was assessed via immunofluorescence (IF) staining, with nuclei stained using DAPI (blue). Scale bar, 5 µm. (D) In vitro GST pull‐down analysis of HEY1 – USP28 interaction. (E) Schematic diagram showing the functional domains of USP28 and HEY1. (F,G) IB and IP products analysis of USP28‐HEY1 interaction in HEK293T cells expressing Flag‐USP28 wild‐type (WT) or the indicated truncated HEY1 mutants (F), or HA‐HEY1 WT or the indicated truncated USP28 mutants (G). (H) Polyubiquitination of HEY1 was assessed by co‐transfecting HEK293T cells with HA‐tagged wild‐type (WT) or truncated HEY1 mutants and GFP‐tagged ubiquitin (GFP‐Ub). Cells were treated with 20 µm MG132 for 8 h before lysis. Immunoblotting was performed on WCL and anti‐HA IPs from these transfected and treated cells. (I) An in vivo ubiquitination assay was performed to assess HEY1 modification in HEK293T cells expressing HA‐tagged wild‐type HEY1 or the indicated truncated mutants, with or without co‐expression of USP28. 36 h post‐transfection, cells were treated with 20 µm MG132 for 8 h before harvest and analysis. (J) IB analysis of the expression levels of specified truncated HEY1 mutants in HEK293T cells transfected with escalating concentrations of Flag‐USP28. (K) Western blot analysis of HEY1 protein stability in transfected HEK293T cells. Following 36 h transfection, cells were exposed to CHX (100 µg/mL) and harvested at indicated times (Left Panel). HEY1 levels were quantified via ImageJ and displayed graphically (Right Panel). (L) In vivo ubiquitination assay in HEK293T cells co‐expressing USP28 and Flag‐HEY1 (WT or K87R). Following MG132 treatment (20 µm, 8 h), HEY1 polyubiquitination was examined by immunoblot analysis. (M) In vivo ubiquitination assays were performed on WCL and anti‐Flag IPs from Huh7 cells expressing the indicated proteins with or without USP28 knockdown. 36 h after transfection, cells were treated with the 20 µm MG132 for 8 h before harvesting. (N) In vivo ubiquitination assays of WCL and anti‐Flag IPs derived from HEK293T cells transfected with plasmids expressing the indicated proteins. 36 h after transfection, cells were treated with the 20 µm MG132 for 8 h before cell collection. (O) IB analysis of indicted proteins obtained from PLC/PRF/5 CSC cells stably expressing HEY1‐WT or ‐K87R mutant with endogenous HEY1 knockdown. (P,Q) Colony and sphere formation were evaluated in PLC/PRF/5 CSCs with shRNA‐mediated endogenous HEY1 knockdown and stable re‐expression of HEY1‐WT or the K87R mutant. ^*^ *p* < 0.05, ^**^ *p* < 0.01 (*t*‐test).(R) Evaluation of subcutaneous tumor formation using PLC/PRF/5 CSCs with endogenous HEY1 knockdown and stable reconstitution of either wild‐type HEY1 (HEY1‐WT) or the K87R mutant. Tumor weights at the study endpoint are presented as mean ± SEM (*n* = 5 per group). ^*^ *p* < 0.05, ^***^ *p* < 0.001, *t*‐test. (S) In vivo tumor growth was measured at the indicated time points and tumors were dissected at the endpoint. Data are presented as mean ± SEM, *n* = 5 per group. (T) A schematic model illustrating the proposed regulatory mechanism through which USP28 modulates HEY1 stability and function.

To further elucidate the molecular mechanism underlying USP28‐mediated deubiquitination of HEY1, we identified USP28 as the sole interacting partner of HEY1 among the DUBs tested (Figure 4A and Figure S4A–C). This interaction was validated in the nucleus by reciprocal co‐immunoprecipitation (Co‐IP) of endogenous USP28 and HEY1, as well as by immunofluorescence (IF) staining (Figure 4B,C). In vitro GST pull‐down assays confirmed a direct physical interaction between USP28 and HEY1 (Figure 4D). Mapping analyses revealed that the N‐terminal domain of USP28 is both necessary and sufficient for binding to either the N‐ or C‐terminal regions of HEY1 (Figure 4E–G).

Protein ubiquitination regulates stability, trafficking, and protein‐protein interactions through seven types of lysine‐linked polyubiquitin chains [30]. To determine the ubiquitin‐linkage specificity of USP28‐mediated HEY1 deubiquitination, we generated lysine (K)‐restricted ubiquitin mutants in which all lysine residues except one were replaced by arginine (R). Comparative analyses with wild‐type ubiquitin revealed that HEY1 is modified by all seven lysine‐linked polyubiquitin chains (K6, K11, K27, K29, K33, K48, and K63) (Figure S4D). Strikingly, ectopic expression of USP28 markedly reduced all seven linkage‐specific ubiquitin chains on HEY1 (Figure S4E), indicating that USP28 exhibits broad linkage promiscuity in HEY1 deubiquitination.

Further mapping showed that the N‐terminal fragment of HEY1 (aa 1–121), particularly the helix‐loop‐helix (HLH) domain, was both necessary and sufficient for HEY1 ubiquitination in cells (Figure 4H). Consistently, removal of polyubiquitin chains was essential for USP28 binding, leading to HEY1 deubiquitination and stabilization (Figure 4I,J). The N‐terminal region of HEY1 (aa 1–121) contains nine lysine residues (K2, K23, K51, K59, K82, K87, K90, K102, and K110) predicted to serve as potential ubiquitination sites (Figure S4F). Mutation of lysine 87 (K87) within the HLH domain to arginine (R) strongly impaired HEY1 polyubiquitination and degradation (Figure S4G) and significantly prolonged its half‐life (Figure 4K). Notably, USP28‐mediated deubiquitination and stabilization of HEY1 were abolished when the K87 residue was mutated (Figure 4L and Figure S4H,I). Sequence alignment revealed that the K87 residue is evolutionarily conserved across species (Figure S4F). Moreover, in USP28‐deficient or AZ‐1‐treated cells, HEY1 polyubiquitination was markedly increased in wild‐type HEY1 but not in the ubiquitination‐deficient K87R mutant (Figure 4M,N and Figure S4J). These findings demonstrate that USP28 specifically deubiquitinates HEY1 at the conserved K87 residue to maintain its stability.

To assess the functional impact of HEY1 deubiquitination on CSC self‐renewal and tumorigenesis, we compared the biological activities of the HEY1 K87R mutant and wild‐type HEY1. The K87R‐mutant cells displayed markedly enhanced sphere‐forming ability, clonogenicity, and tumorigenic potential relative to wild‐type controls (Figure 4O–S). Collectively, these data indicate that USP28‐mediated removal of polyubiquitin chains at lysine 87 stabilizes HEY1 and potentiates its oncogenic activity in vitro and in vivo (Figure 4T).

### 2.5 IKKβ‐mediated Phosphorylation of HEY1 at Ser40 Promotes USP28 Binding and Stabilizes HEY1

**FIGURE 5:**
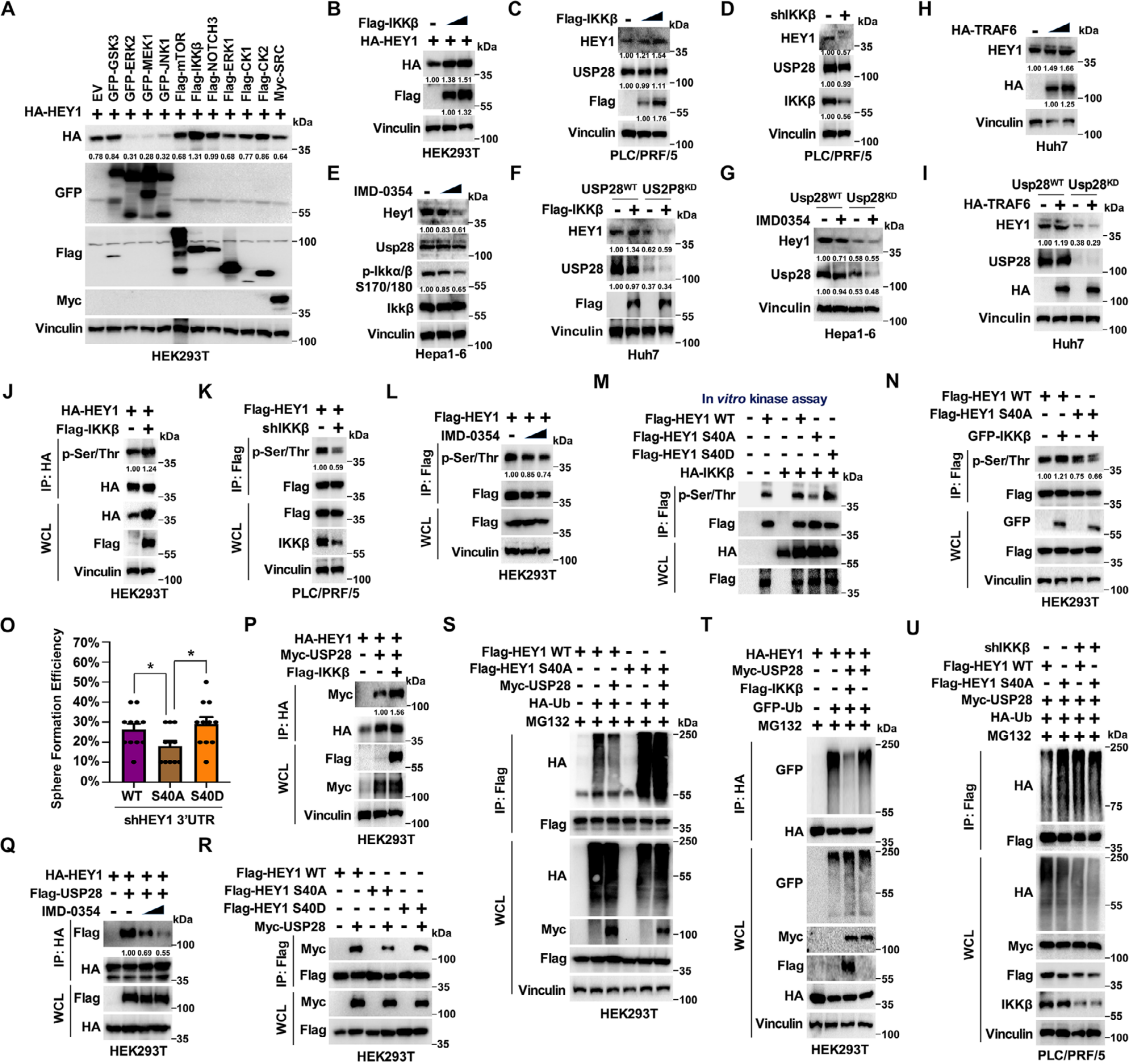
IKKβ phosphorylates HEY1 at Ser40 to promote USP28 recruitment and HEY1 stabilization. (A) IB analysis of WCL derived from HEK293T cells co‐transfected HA‐HEY1 with different kinases as indicated. (B,C) HEY1 protein abundance was assessed by western blot in PLC/PRF/5 or HEK293T cells expressing increasing quantities of Flag‐tagged IKKβ. (D) IB analysis of WCL from PLC/PRF/5 cells with IKKβ knockdown by shRNA. (E) IB analysis of Hepa1‐6 cells treated with graded concentrations of the IKKβ inhibitor IMD‐0354 (24 h) using the antibodies indicated. (F) Knockdown of USP28 inhibits IKKβ‐induced accumulation of HEY1. The WT and USP28‐KD Huh7 cells were transfected with Flag‐IKKβ for 36 h before they were harvested. (G) WT and Usp28‐KD cells were exposed to IMD‐0354 (10 µm) for 36 h prior to collection and subsequent IB analysis. (H) IB analysis of HEY1 protein levels in Huh7 cells expressing increasing amount of HA‐TRAF6. (I) The WT and USP28‐KD Huh7 cells were transfected with HA‐TRAF6 for 36 h before they were harvested. (J) An in vivo phosphorylation assay was performed to evaluate the phosphorylation status of HA‐HEY1 in HEK293T cells with or without overexpression of Flag‐IKKβ. (K) In vivo phosphorylation assay of Flag‐tagged HEY1 in IKKβ‐KD PLC/PRF/5 cells. (L) In vivo phosphorylation assay of Flag‐tagged HEY1 in HEK293T cells treated with increasing concentrations of IKKβ inhibitor IMD‐0354 for 24 h before harvesting. (M) In vitro kinase assays were performed with purified active IKKβ, WT HEY1and HEY1 mutants (S40A or S40D). (N) IB analysis of lysates and IP samples from HEK293T cells co‐expressing GFP‐IKKβ and Flag‐HEY1 constructs after 36 h of transfection. (O) Sphere formation assays of PLC/PRF/5 CSC cells with stably expressing the indicated HEY1 constructs with endogenous HEY1 knockdown by shRNA. ^*^ *p* < 0.05, *t*‐test. (P) IB analysis was performed on WCL and IP samples derived from HEK293T cells transfected with the indicated constructs. (Q) HEK293T cells were transfected with specified plasmids for 24 h, followed by a 24 h pretreatment with increasing doses of IMD‐0354. WCL and IP were then prepared for immunoblot analysis. (R) IB analysis was conducted on WCL and IP samples from HEK293T cells co‐transfected with various Flag‐tagged HEY1 constructs and Myc‐tagged USP28. (S) To assess HEY1 ubiquitination in vivo, transfected HEK293T cells were exposed to 20 µm MG132 for 8 h after 36 h of expression and subsequently harvested for analysis. (T) Effects of IKKβ on USP28‐mediated HEY1 polyubiquitination. HEK293T cells were transfected with the indicated plasmids. HA‐tagged HEY1 was immunoprecipitated with an anti‐HA antibody and analyzed by immunoblotting to detect ubiquitination. (U) In vivo ubiquitination assay of HEY1 in WT or IKKβ‐KD PLC/PRF/5 cells expressing indicated constructs. Cells were harvested after 36 h of transfection and an 8 h treatment with 20 µm MG132.

Phosphorylation and ubiquitination are two major post‐translational modifications whose interplay critically determines protein fate and function [31]. Our recent study demonstrated that CK1‐mediated phosphorylation of SOX9 at Thr196 is required for its ubiquitination and subsequent tumor suppression [32]. Similarly, IKKβ‐mediated phosphorylation of GLI1 has been shown to enhance its deubiquitination and stabilization [33]. Previous mass spectrometry datasets identified multiple kinases as potential HEY1‐interacting partners (https://thebiogrid.org). To identify kinases that regulate HEY1 protein abundance, we screened a panel that included CK2 and NOTCH, both listed among predicted interactors, as well as several other key signaling kinases, such as IKKβ, AKT isoforms, and ERK (Figure 5A and Figure S5A). Among the kinases tested, IKKβ was the most potent in increasing both endogenous and exogenous HEY1 protein levels, while USP28 expression remained unchanged (Figure 5A–C and Figure S5A–C). Co‐IP and truncation analyses revealed that IKKβ directly interacts with HEY1 and preferentially stabilizes its N‐terminal domain (aa 1–121) (Figure S5D,E). The IKKβ‐mediated increase in HEY1 abundance was largely attributed to an extended protein half‐life (Figure S5F,G). Conversely, genetic silencing of IKKβ or pharmacological inhibition using IMD‐0354 significantly reduced HEY1 levels and shortened its half‐life, without affecting USP28 expression (Figure 5D,E and Figure S5H–K). Clinically, higher IKKβ expression in tumor tissues correlated with poorer patient survival (Figure S5L), while IKKβ depletion markedly suppressed tumorigenesis and HEY1 expression in vivo (Figure S5M–O). Strikingly, depletion of USP28 completely abolished both the IKKβ‐mediated upregulation of HEY1 and its downregulation upon IMD‐0354 treatment (Figure 5F,G and Figure S5P), suggesting that IKKβ stabilizes HEY1 in a USP28‐dependent manner. Similarly, ectopic expression of the IKKβ upstream activator TRAF6 [34] increased HEY1 protein abundance (Figure 5H and Figure S5Q), an effect that was fully abrogated by USP28 deficiency (Figure 5I). These findings indicate that activation of the IKKβ signaling pathway promotes HEY1 stabilization through USP28.

Given that IKKβ functions as a serine kinase, we hypothesized that HEY1 is a direct substrate for IKKβ‐mediated phosphorylation. Indeed, overexpression of IKKβ enhanced HEY1 phosphorylation (Figure 5J), whereas IKKβ KD or IMD‐0354 treatment markedly reduced it (Figure 5K,L). In vitro kinase assays further confirmed that IKKβ directly phosphorylates HEY1 (Figure 5M). Immunoprecipitation‐mass spectrometry (IP‐MS) analysis identified four potential phosphorylation sites‐Ser40, Ser139, Ser156, and Ser221‐upon ectopic IKKβ expression (Figure S6A,B). Mutation of Ser40 to alanine (S40A), which prevents phosphorylation, completely abolished USP28‐mediated HEY1 stabilization (Figure S6C). In contrast, the phospho‐mimetic mutant HEY1‐S40D (Ser40→Asp) displayed a markedly prolonged half‐life compared with HEY1‐S40A (Figure S6D). Consistent with this, HEY1‐S40D, but not S40A, exhibited increased phosphorylation upon IKKβ overexpression and decreased phosphorylation following IMD‐0354 treatment (Figure 5M and Figure S6E). Functionally, HEY1‐S40A‐expressing cells showed significantly reduced sphere‐forming capacity compared with wild‐type HEY1 (Figure 5O and Figure S6F), underscoring the functional importance of Ser40 phosphorylation.

We next examined whether IKKβ‐mediated phosphorylation influences HEY1's interaction with USP28. Co‐IP assays revealed that IKKβ overexpression strengthened the HEY1‐USP28 interaction (Figure 5P), whereas IMD‐0354 treatment markedly weakened it (Figure 5Q and Figure S6G). Consistently, the phosphorylation‐deficient HEY1‐S40A mutant exhibited diminished binding to USP28 (Figure 5R and Figure S6H). These results demonstrate that phosphorylation of HEY1 at Ser40 by IKKβ is required for its association with USP28.

Given that USP28 deubiquitinates and stabilizes HEY1 (Figure 3), we next assessed how IKKβ‐mediated phosphorylation affects HEY1 ubiquitination. Compared with wild‐type HEY1, the HEY1‐S40A mutant was heavily ubiquitinated and resistant to USP28‐mediated deubiquitination (Figure 5S and Figure S6I). Ectopic IKKβ expression enhanced USP28‐dependent HEY1 deubiquitination, whereas IMD‐0354 treatment suppressed it (Figure 5T and Figure S6J). Furthermore, genetic depletion of IKKβ significantly increased endogenous HEY1 ubiquitination (Figure 5U). Collectively, these results demonstrate that IKKβ directly phosphorylates HEY1 at Ser40 to promote its interaction with USP28, thereby facilitating HEY1 deubiquitination and stabilization.

### 2.6 USP28 Deficiency Triggers Tumor Cell‐Intrinsic Immune Responses to Remodel Anti‐Tumor Immunity

**FIGURE 6:**
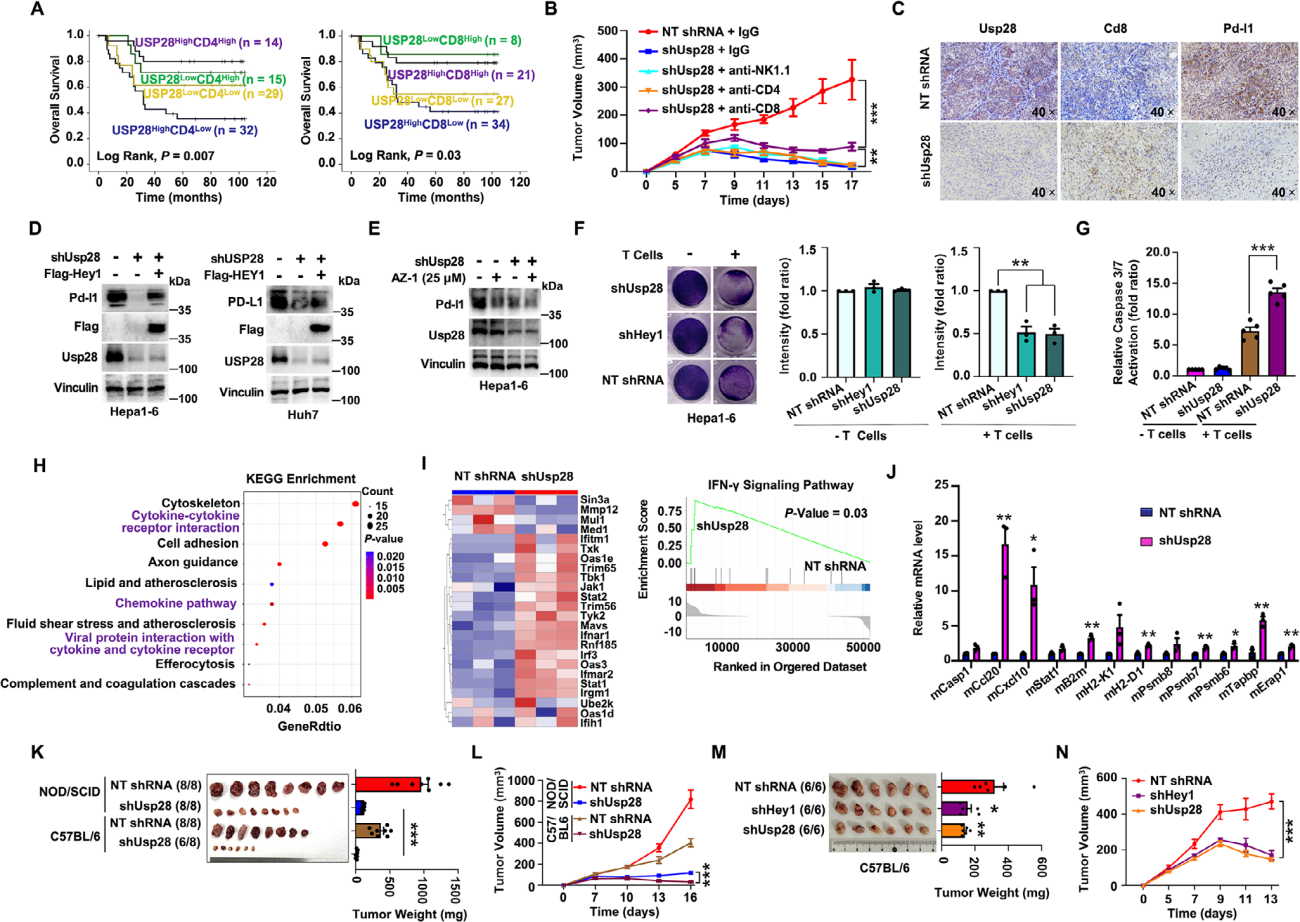
USP28 deficiency attenuates PD‐L1 expression and tumor‐intrinsic immunosuppression to enhance anti‐tumor immunity. (A) Kaplan‐Meier survival curves showing the association of USP28/CD4 and USP28/CD8 co‐expression with overall survival in 90 patients with hepatocellular carcinoma (HCC). (B) WT or Usp28‐KD Hepa1‐6 cells (2.5 × 10^6^) were subcutaneously injected into C57BL/6 mice that had been pre‐treated with antibodies against immunoglobulin G (IgG), CD8, CD4 and NK1.1 (*n* = 8 per group). Tumor growth was tracked by measuring tumor dimensions every other day, starting from day five post‐inoculation. ^**^ *p* < 0.01, ^***^ *p* < 0.001. (C) Loss of Usp28 leads to decreased Pd‐l1 protein levels in the xenograft tumors. Representative IHC images of Pd‐l1 and Cd8 in tumor sections from control and Usp28‐depleted Hepa1‐6 xenografts. (D) IB analysis of PD‐L1 in the WT and Usp28‐KD Hepa1‐6 and Huh7 cells transfected with Flag‐HEY1 for 36 h before harvesting. (E) IB analysis of Pd‐l1 in the WT and Usp28‐KD Hepa1‐6 cells treated with 10 µm USP28 inhibitor AZ‐1 for 24 h before harvesting. (F) A T cell‐mediated cytotoxicity assay was performed using Hepa1‐6 cells with knockdown of either Hey1 or Usp28. Activated T cells were co‐cultured with tumor cells in 24‐well plates for three days, after which surviving adherent tumor cells were stained with crystal violet and visualized. The relative fold change in surviving cell intensity is presented. ^**^ *p* < 0.01. (G) Caspase‐3/7 activation was assessed in a 24 h co‐culture of activated T cells and Usp28‐KD Hepa1‐6 cells in 96‐well plates. Results represent mean ± SEM of three independent experiments (^***^ *p* < 0.001, two‐tailed unpaired *t*‐test). (H) Gene Ontology (GO) analysis revealed the top 10 functional terms associated with immune‐related genes that were transcriptionally repressed in Usp28‐deficient Hepa1‐6 cells (*n* = 3 biological replicates). Enrichment significance was determined using modified Fisher's exact tests. (I) Differential gene expression within the IFN‐γ signaling pathway was visualized via gene‐set enrichment analysis (GSEA) and a heatmap, generated from triplicate biological samples per group. (J) RT‐qPCR analysis of indicated gene expression in Hepa1‐6 cells with shRNA‐mediated Usp28 knockdown vs. NT shRNA control. *n*  =  3. ^*^ *p* < 0.05, ^**^ *p* < 0.01, *t*‐test. (K,L) Usp28 knockdown in Hepa1‐6 cells (2.5 × 10^6^) suppresses tumor growth in both NOD/SCID xenografts and C57BL/6 syngeneic models. Mice were sacrificed 16 days after implantation. Tumor growth over time (L). Tumor image and tumor weight are presented (K). Data are presented as mean ± SEM. ^***^ *p* < 0.001. (M,N) A total of 5.0 × 10^6^ Hepa1‐6 cells (WT or Hey1‐KD) were subcutaneously implanted in C57BL/6 mice. Tumor growth over time (N). Tumor image and tumor weight are presented (M). Data are presented as mean ± SEM. ^*^ *p* < 0.05, ^**^ *p* < 0.01, ^***^ *p* < 0.001.

Emerging evidence indicates that CSCs facilitate tumor immune evasion primarily by suppressing CD8^+^ cytotoxic T lymphocyte (CTL) infiltration into the tumor microenvironment. We therefore asked whether inhibition of USP28 in tumors could enhance immune‐cell infiltration and suppress HCC progression. Analysis of Tumor Immune Dysfunction and Exclusion (TIDE) datasets [35] revealed that higher CTL levels correlated with improved survival in HCC cases exhibiting low USP28 expression, but not in those with high USP28 expression (Figure S7A). Consistently, The Cancer Genome Atlas (TCGA) data showed a strong negative correlation between USP28 expression and CD8^+^ T‐cell infiltration in HCC (Figure S7B). Immunohistochemical (IHC) staining of 90 HCC specimens, together with TCGA analysis, further demonstrated that higher densities of CD4^+^ and CD8^+^ tumor‐infiltrating lymphocytes (TILs) predicted better survival only in tumors with low USP28 expression (Figure 6A and Figure S7C). To identify the immune subset responsible for USP28‐mediated tumor control, we depleted NK cells, CD4^+^ T cells, or CD8^+^ T cells using neutralizing antibodies in Hepa1‐6 syngeneic tumor models. Depletion of CD8^+^ T cells, but not CD4^+^ T cells or NK cells, abrogated the growth difference between wild‐type and Usp28‐KD tumors (Figure 6B), demonstrating that USP28 promotes tumor evasion from CD8^+^ T‐cell‐mediated immune surveillance in HCC.

Because PD‐L1 upregulation in CSCs is known to drive immune suppression by impairing CD8^+^ T‐cell trafficking and cytotoxic function [36, 37, 38], we next examined whether USP28 influences PD‐L1 expression. Bioinformatic analysis (GEPIA, http://gepia2.cancer‐pku.cn/#index) revealed a positive correlation between USP28 and PD‐L1 expression (Figure S7D). Consistently, tumors with Usp28 depletion displayed lower PD‐L1 levels and increased CD8^+^ T‐cell infiltration (Figure 6C). Mechanistically, USP28 positively regulated PD‐L1 expression in a HEY1‐dependent manner (Figure 6D and Figure S7E–G). Pharmacological inhibition of USP28 with AZ‐1 reduced PD‐L1 expression in a dose‐ and time‐dependent manner (Figure 6E and Figure S7H–L). Interestingly, USP28 deficiency or AZ‐1 treatment selectively decreased PD‐L1 abundance in cytosolic and membrane compartments, without affecting its nuclear localization (Figure S7M–P). In co‐culture assays, depletion of Usp28 or Hey1 in Hepa1‐6 cells markedly enhanced T‐cell cytotoxicity (Figure 6F,G and Figure S8A), consistent with the results obtained following AZ‐1 treatment (Figure S8B–E). Together, these findings indicate that the USP28‐HEY1 axis promotes PD‐L1‐mediated immunosuppression, thereby dampening CD8^+^ T‐cell activity. To gain a global view of USP28‐regulated immune pathways, we performed transcriptomic profiling of Usp28‐deficient Hepa1‐6 cells. RNA‐seq analysis revealed widespread transcriptional reprogramming (Figure 6H and Figure S8F), with significant upregulation of immune‐related pathways, particularly those associated with interferon‐γ (IFNγ) signaling, chemokine responses, and innate immune activation (Figure 6I and Figure S8G). RT‐qPCR validation confirmed increased expression of IFNγ‐responsive genes following USP28 loss (Figure 6J). Notably, Usp28 depletion also led to enrichment of PD‐1 response‐associated gene signatures (Figure S8H).

Further analysis revealed enrichment of MHC‐I antigen‐processing and presentation gene sets in Usp28‐deficient cells (Figure S8I), which was validated by RT‐qPCR (Figure 6J). Consistent with these results, bioinformatic data showed that MHC‐I pathway genes were significantly upregulated in liver adenocarcinoma patients with low USP28 expression compared to those with high expression (Figure S8J). Moreover, AZ‐1 treatment increased cell‐surface H2K^d^/H2D^d^ levels, whereas re‐expression of wild‐type or K87R‐mutant HEY1 abolished this effect (Figure S8K–M). These results support a model in which reduced USP28 or HEY1 activity induces immune‐stimulatory gene programs‐including IFNγ signaling, chemokine production, and antigen presentation‐thereby enhancing cytotoxic T‐cell recruitment and activation.

Finally, to evaluate the in vivo relevance of these findings, we implanted Hepa1‐6 cells into immunocompetent and immunodeficient mice. Depletion of Usp28 or Hey1 resulted in significantly greater tumor growth inhibition in immunocompetent hosts compared with immunodeficient hosts (Figure 6K–N and Figure S8N,O). These results indicate that loss of USP28 or HEY1 primarily augments tumor‐cell‐intrinsic activation of anti‐tumor T‐cell immunity rather than affecting intrinsic tumor cell proliferation, thereby restraining HCC progression.

### 2.7 Targeting USP28 Potentiates Anti‐PD‐1 Immunotherapy in Murine HCC Models

**FIGURE 7:**
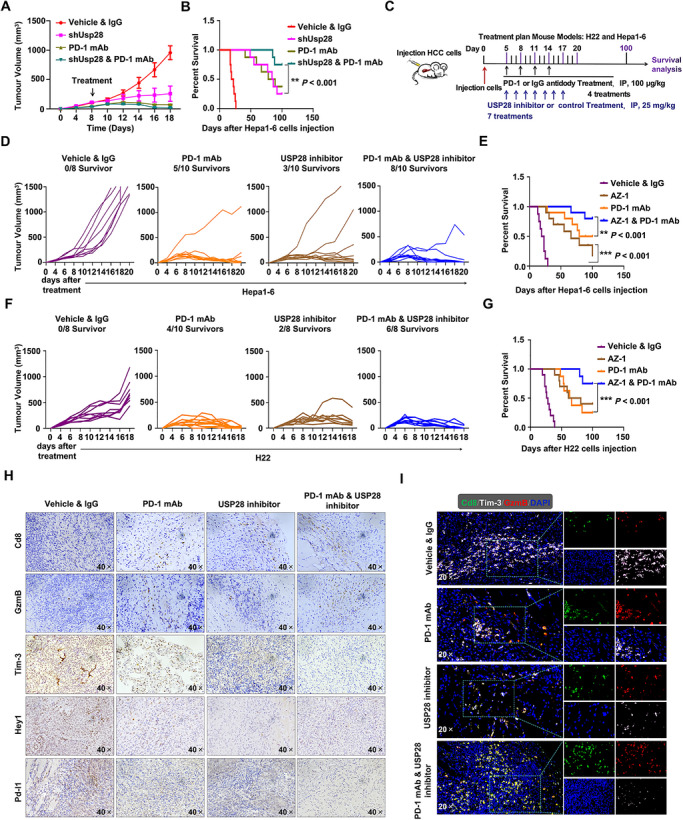
Targeting USP28 enhances the efficacy of anti‐PD‐1 immunotherapy in murine HCC models. (A) Growth of Usp28‐knockdown Hepa1‐6 tumors was assessed in C57BL/6 mice administered control IgG or anti‐PD‐1 antibody (*n* = 10 per group). (B) Kaplan‐Meier survival analysis across four treatment groups indicates that depletion of Usp28 enhances the therapeutic efficacy of PD‐1mAb treatment. ^**^ *p* < 0.01. (C) Schematic representation of the in vivo treatment strategy. Subcutaneous Hepa1‐6 or H22 tumors in male C57BL/6 mice were treated with either anti‐IgG control, anti‐PD‐1 mAb, USP28 inhibitor AZ‐1, or the combination of anti‐PD‐1 and AZ‐1. (D,F) Mice bearing Hepa1‐6 or H22 subcutaneous tumors were assigned to the indicated treatment groups. Tumor volumes in animals receiving control antibody, anti‐PD‐1 monoclonal antibody, the USP28 inhibitor AZ‐1, or combination therapy were measured every two days and plotted individually, with *n* = 10 mice per group. (E,G) Kaplan‐Meier survival analysis across treatment groups reveals that the combination of PD‐1 monoclonal antibody and the USP28 inhibitor AZ‐1 significantly enhances therapeutic efficacy ^**^ *p* < 0.01. (H) Immunohistochemical (IHC) staining was performed to evaluate the expression levels of Cd8, Granzyme B (GzmB), Tim‐3, Pd‐l1, and Hey1 in Hepa1‐6 tumor tissues following the indicated treatments. (I) Multiplex IHC staining of Hepa1‐6 tumors after treatment shows Cd8 (green), GzmB (white), Tim‐3 (red), and DAPI (blue) for nuclear visualization.

Based on our mechanistic findings, we hypothesized that inhibition of USP28 could synergize with immune checkpoint blockade to elicit enhanced anti‐tumor immunity. Indeed, genetic depletion of Usp28 markedly sensitized Hepa1‐6 tumors to anti‐PD‐1 immunotherapy in immunocompetent C57BL/6 mice (Figure 7A,B). To further assess the translational potential of pharmacologic targeting, we evaluated the efficacy of combining the USP28 inhibitor AZ‐1 with anti‐PD‐1 therapy in a syngeneic murine HCC model (Figure 7C). Compared with either monotherapy, combined AZ‐1 and anti‐PD‐1 treatment significantly suppressed tumor growth and markedly prolonged overall survival (Figure 7D–G). Analysis of infiltrated immune cells demonstrated that the USP28 inhibitor combined with anti‐PD‐1 treatment significantly increased the percentage of CD8^+^ T cells (Figure 7H,I and Figure S9A). To determine whether USP28 inhibition also modulates CD8^+^ T‐cell functional states, we assessed markers of activation and exhaustion. The combination treatment significantly elevated expression of the cytotoxic effector molecule Granzyme B (GzmB) while reducing the exhausted T‐cell marker Tim‐3 in tumor‐infiltrating CD8^+^ T cells (Figure 7H,I and Figure S9A). Consistent with our proposed mechanism, tumors treated with AZ‐1 or the combination regimen exhibited substantial downregulation of Pd‐l1 and Hey1 expression compared with controls (Figure 7H). Together, these findings demonstrate that USP28 inhibition enhances the efficacy of anti‐PD‐1 immunotherapy by augmenting CD8^+^ T‐cell infiltration and activation, thereby promoting robust anti‐tumor immunity. Collectively, our results identify pharmacologic inhibition of USP28‐alone or in combination with immune checkpoint blockade‐as a promising therapeutic strategy for hepatocellular carcinoma.

### 2.8 USP28 Expression is Inversely Correlated With Response to PD‐1 Blockade in Human Tumors

**FIGURE 8:**
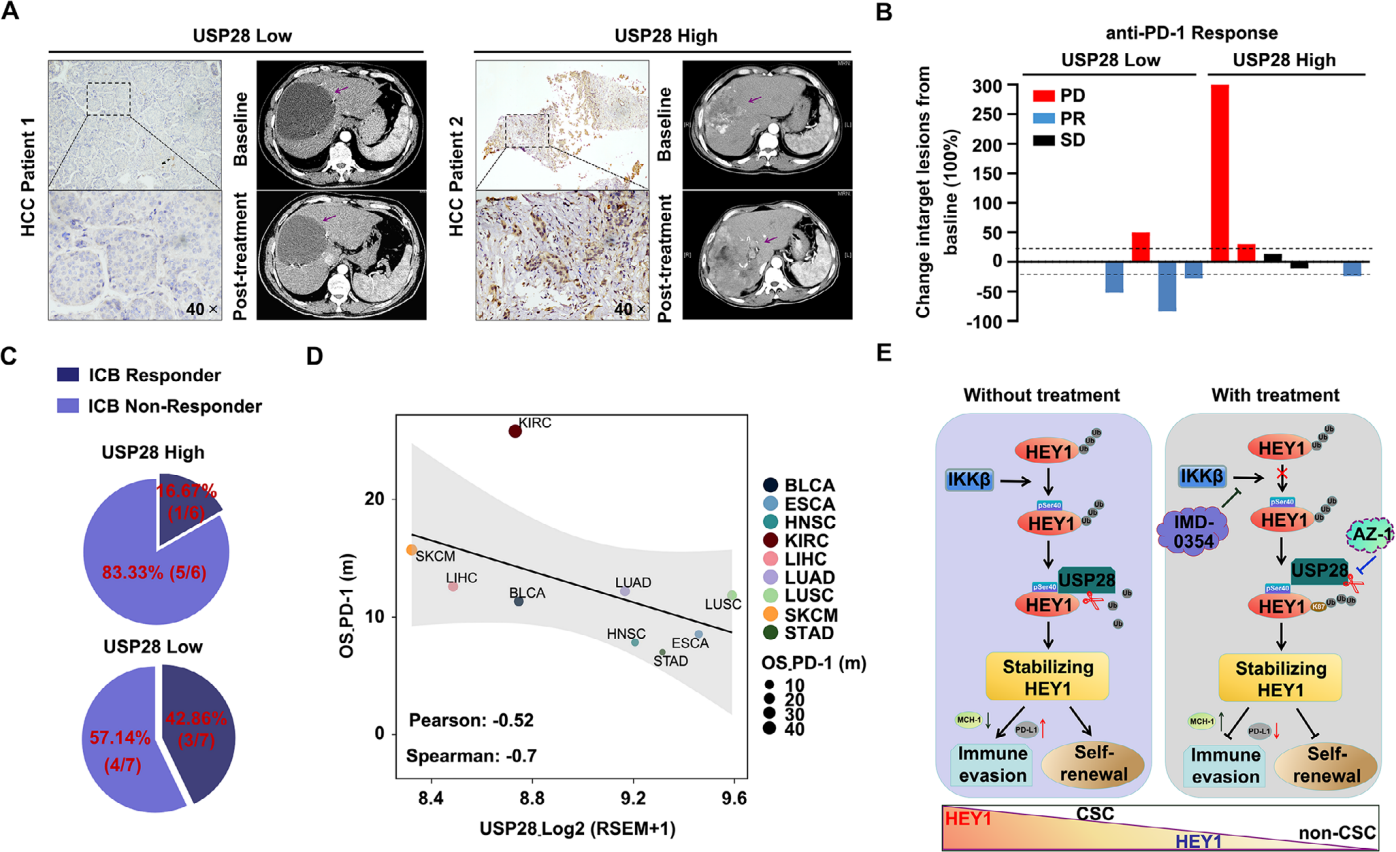
USP28 expression predicts response to anti‐PD‐1 immunotherapy. (A) Representative immunohistochemical staining for USP28 protein in tumor tissues (top: 10× magnification, bottom: 40× magnification) alongside corresponding CT images from hepatocellular carcinoma (HCC) patients receiving anti‐PD‐1/PD‐L1 therapy are presented. (B) A waterfall plot illustrates the antitumor response to anti‐PD‐1 therapy, measured as the maximum reduction in total target lesion size relative to baseline, in cancer patients stratified by low or high USP28 expression. Individual bars represent each patient, with color coding indicating treatment outcome: partial response (PR), stable disease (SD), or progressive disease (PD). Dashed black lines denote RECIST 1.1‐defined response thresholds. (C) Pie charts showing the response rates of HCC patients with low or high USP28 expression. (D) Overall survival following PD‐1 monoclonal antibody (mAb) treatment was analyzed among cancer patients with USP28 expression levels using the ImmunoCheckDB dataset. (https://smuonco.shinyapps.io/ImmunoCheckDB/). (E) A mechanistic model for targeting USP28‐HEY1 axis overcomes PD‐1/PD‐L1 blockade resistance in HCC by suppressing PD‐L1 expression and immunosuppression. USP28 upregulates PD‐L1, promoting immune evasion and driving therapy resistance. Inhibition of USP28 with AZ‐1 downregulates PD‐L1 and attenuates immunosuppression, thereby restoring the efficacy of anti‐PD‐1/PD‐L1 immunotherapy.

To directly evaluate the relationship between USP28 expression and clinical responses to PD‐1 blockade in HCC patients, we analyzed data from a cohort of individuals enrolled in a basket trial of anti‐PD‐1 therapy [19]. Patients were stratified into two groups based on tumoral USP28 expression levels. Strikingly, patients who achieved a partial response (PR) or stable disease (SD) exhibited significantly lower USP28 expression compared with those who experienced progressive disease (PD) (Figure 8A,B). Consistently, the overall response rate (ORR) was markedly higher in patients with low USP28 expression than in those with high USP28 levels (42.86% vs. 16.67%) (Figure 8C).

To assess the broader translational relevance of these findings, we further examined publicly available datasets across multiple human cancer types. High USP28 expression was consistently associated with significantly shorter overall survival compared with low USP28 expression (Figure S9B). In addition, elevated CTL infiltration correlated with improved survival in tumors exhibiting low USP28 expression but not in those with high USP28 expression (Figure S9C). Importantly, higher USP28 expression was linked to poorer overall survival and a reduced likelihood of clinical benefit from PD‐1 antibody therapy (Figure 8D). Collectively, these results demonstrate that tumoral USP28 expression inversely correlates with response to PD‐1 blockade and overall patient survival, underscoring the therapeutic potential of USP28 inhibition to enhance the efficacy of immune checkpoint immunotherapy in HCC and potentially other cancers.

## 3 Discussion

The differentiation and trans‐differentiation of CSCs are precisely orchestrated by multiple interdependent extracellular cues and cell‐intrinsic signaling pathways, among which ubiquitination has emerged as a critical regulatory mechanism that governs protein turnover [39, 40, 41]. In this study, we identified a previously unrecognized IKKβ‐USP28‐HEY1 signaling axis that controls CSC maintenance and tumor immune evasion in HCC. We demonstrated that USP28 directly interacts with and deubiquitinates HEY1, thereby stabilizing HEY1 and suppressing CSC differentiation. In parallel, IKKβ phosphorylates HEY1 at Ser40, promoting its association with USP28 and further enhancing HEY1 stabilization. Functionally, this axis acts as a tumor cell‐intrinsic checkpoint that limits anti‐tumor immunity, positioning USP28 as a promising therapeutic target for immunomodulation. Pharmacological inhibition of USP28 potentiated the efficacy of PD‐1 immune checkpoint blockade (ICB) in murine HCC models, highlighting the translational potential of USP28‐targeted therapies as rational combination partners for ICB regimens (Figure 8E).

Previous studies have implicated HEY1 as a key driver of tumorigenesis and metastasis across various malignancies [9, 10, 11, 12, 42]. Our findings extend these observations by establishing HEY1 as a central regulator of liver CSC self‐renewal and tumorigenic potential. Silencing HEY1 markedly impaired CSC proliferation and sphere formation, suggesting that targeting HEY1 may represent a therapeutic approach for eradicating CSCs in HCC. Mechanistically, our data reveal that dynamic HEY1 ubiquitylation governs CSC differentiation. USP28 stabilizes HEY1 via direct deubiquitination, independent of transcriptional regulation, while IKKβ‐mediated phosphorylation at Ser40 facilitates the USP28‐HEY1 interaction and reinforces protein stability. Collectively, we propose a dynamic stabilization cascade in which IKKβ and USP28 cooperatively regulate HEY1 post‐translationally to control CSC fate and initiate tumor growth. In addition, our preliminary data suggest that Cullin 1 may antagonize USP28‐mediated HEY1 stabilization (unpublished observations), raising the possibility that specific cullin‐based E3 ligases counterbalance this pathway to promote HEY1 degradation and CSC differentiation.

Although aberrant USP28 expression has been reported in multiple human cancers, where it promotes proliferation and tumor progression [43, 44, 45], its role in tumor immunity and immunotherapy response has remained unclear. Our results demonstrate that USP28 deficiency triggers robust anti‐tumor immune activation characterized by increased CD8^+^ T‐cell infiltration, enhanced immunogenicity, and suppressed tumor growth. Mechanistically, the USP28‐HEY1 axis regulates PD‐L1 expression and modulates T‐cell activity, thereby linking CSC maintenance to immune evasion. While PD‐1/PD‐L1 blockade has provided clinical benefit in several advanced cancers, the response rate to ICB monotherapy in HCC remains modest (∼20%) [46, 47]. We show that the USP28‐HEY1 axis not only sustains CSC properties but also upregulates PD‐L1 expression, collectively impairing anti‐tumor immunity by reducing MHC‐I‐mediated antigen presentation and reinforcing an immunosuppressive tumor microenvironment (TME). Importantly, both genetic ablation and pharmacological inhibition of USP28 synergized with PD‐1 blockade to suppress tumor progression and prolong survival across multiple murine models (Figure 7). These findings provide a compelling rationale for combining USP28 inhibition with ICB in future clinical studies and for developing biomarkers to identify tumors most likely to benefit from this approach. Future work using patient‐derived models, including organoids (PDOs) and xenografts (PDXs), will be essential to validate this signaling axis and accelerate clinical translation.

In summary, our work uncovers a novel post‐translational regulatory mechanism in which IKKβ‐mediated phosphorylation and USP28‐dependent deubiquitination cooperatively stabilize HEY1, thereby maintaining CSC stemness and promoting immune evasion. This study highlights the dual oncogenic and immunosuppressive roles of the USP28‐HEY1 axis and provides proof‐of‐concept evidence supporting the use of USP28 inhibitors to potentiate PD‐1‐based immunotherapy. Targeting USP28 reprograms the tumor immune microenvironment by enhancing CD8^+^ T‐cell infiltration and activation, offering a promising combinatorial strategy for achieving durable therapeutic responses in HCC and potentially other solid tumors.

## 4 Methods

### 4.1 Cell Culture, Transfection, Lentiviral and Drug Treatments

Human embryonic kidney (HEK) 293T, PLC/PRF/5, Huh7, Hep3B, SK‐HEP‐1, and Hepa1‐6 were cultured in Dulbecco's Modified Eagle's Medium (DMEM); H22 cells were maintained in DMEM/F12 culture medium containing 10% FBS, along with penicillin (100 U/mL) and streptomycin (100 µg/mL). Incubation was carried out at 37°C in a humidified sterile incubator with a 5% CO_2_ environment. All cell lines tested negative for mycoplasma contamination. Upon receipt, the cell lines were authenticated using short tandem repeat (STR) profiling, expanded, cryopreserved, and revived from these stocks every 3 to 4 months. DNA transfection was carried out with the Neofect reagent (Neofect) according to the manufacturer's protocol. Lentiviruses were generated by cotransfecting HEK293T cells using calcium phosphate precipitation, employing a combination of the lentiviral vector and the packaging plasmids pMD2.G, pRSV‐Rev, and pMDLG/pRRE. The collected viral particles were then used for infection, followed by puromycin selection (1 µg/mL).

For drug treatments, MG132 (S2619), AZ‐1 (S8904), and IMD‐0354 (S2864) were purchased from Selleck Chemicals. Cycloheximide (CHX) (N11534) was purchased from Sigma‐Aldrich. Cells were then cultured in medium containing the drugs at the specified concentrations for the time intervals indicated in the text.

### 4.2 Plasmids and shRNAs

Expression vectors GFP‐Ubiquitin (Ub), HA‐Ub (WT), HA‐Ub (K6) only, HA‐Ub (K11) only, HA‐Ub (K27) only, HA‐Ub (K29) only, HA‐Ub (K33) only, HA‐Ub (K48) only, HA‐Ub (K63) only, Flag‐CK1, Flag‐CK2, and Flag‐HA tagged USP family members of DUBs constructs were described previously [19]. GFP‐GSKβ (29680), GFP‐ERK2 (37145), GFP‐MEK1 (14769), GFP‐JNK1 (86830), Flag‐mTOR (26603), Flag‐IKKβ (23298), Flag‐NOTCH3 (40640), Flag‐ERK1 (49328), HA‐CDK1 (27652), HA‐ALK2 (80870), HA‐14‐3‐3 (13270), Flag‐TRAF6 (66929) and HA‐ALK3 (80873) were purchased from Addgene. Flag‐HEY1, HA‐HEY1, Flag‐Hey1, HA‐USP25, HA‐USP28, Flag‐USP28, Myc‐USP28, Flag‐Usp28, HA‐TRAF6, Myc‐SRC, GFP‐ERK2, GFP‐IKKβ and HA‐Usp28 were constructed by cloning the corresponding complementary DNA into the pcDNA3.1 or pCMV vector. HA‐HEY1 (aa 1–121), HA‐HEY1 (aa 122–304), Flag‐USP28 (aa 1–163), Flag‐USP28 (aa 160–652), Flag‐USP28 (aa 1–652), and Flag‐USP28 (aa 563–1077) were generated by sub‐cloning the corresponding cDNAs into the pcDNA3.1 vector. Flag‐HEY1 constructs (K2R, K23R, K51R, K59R, K82R, K87R, K90R, K102R, K110R, S40A, S40D, S139A, S156A, S221A), Flag‐HEY1 K87R, Flag‐Hey1 K87R and Flag‐USP28 C171A mutant were introduced via site‐directed mutagenesis utilizing the QuickChange Q5 Kit (NEBaseChanger) according to the recommended protocol. For gene knockdown, pLKO.1‐puro lentiviral MISSION shRNA constructs targeting the endogenous genes were applied. pLKO‐shUSP28 (TRCN0000007742, TRCN0000007743), pLKO‐shUsp28 (TRCN00000308680), shHEY1 (TRCN0000020217, TRCN0000274312), pLKO‐shHey1 (TRCN0000086482), pLKO‐shIKKβ (TRCN0000018917) and a non‐targeting (NT) control shRNA (TRC1/1.5) were from Sigma‐Aldrich. pLenti‐HA‐HEY1 WT, pLenti‐HA‐HEY1 K87R, pLenti‐Flag‐USP28 and pLenti‐Flag‐Usp28 were generated in this study. All constructs were verified by DNA sequencing. Further details regarding plasmid construction are available upon request.

### 4.3 Antibodies

The antibodies utilized for immunoblotting (IB) in this study are listed below. Anti‐Vinculin (1:2000 dilution, Cat# V9131, RRID:AB_477629) and the Flag‐Tag antibody (1:2000 dilution, Cat# F1804, RRID:AB_262044) were acquired from Sigma‐Aldrich. The following antibodies, all used at a 1:1000 dilution unless otherwise noted, were sourced from Cell Signaling Technology: Anti‐HA‐Tag (Cat# 3724, RRID:AB_1549585), anti‐T7‐Tag (Cat# 13246, RRID:AB_2798161), anti‐Myc‐Tag (Cat# 2278, RRID:AB_490778), anti‐GFP‐Tag (Cat# 2956, RRID:AB_1196615), anti‐His‐Tag (Cat# 12698, RRID:AB_2744546), anti‐ubiquitin (Cat#3936, RRID:AB_331292), anti‐PD‐L1 (Cat# 13684, RRID:AB_2687655), anti‐DYKDDDDK (Cat# 14793, RRID:AB_2572291), anti‐Lamin B1 (Cat# 13435, RRID:AB_2737428), anti‐IKKβ (Cat# 8943), Rabbit (DA1E) mAb IgG XP Isotype Control (Cat# 3900, RRID:AB_1550038), Mouse (G3A1) mAb IgG1 Isotype Control (Cat# 5415, RRID:AB_10829607), anti‐phospho‐IKKα/β (ser176/180) (Cat# 2687, RRID:AB_330566), anti‐GAPDH (Cat# 2118, RRID:AB_561053). Additional primary antibodies and their suppliers are as follows: anti‐ubiquitin (1:1000 dilution, Cat# sc‐8017, RRID:AB_628423) and anti‐c‐MYC (1:1000 dilution, Cat# 631206, RRID:AB_2928131) from Clontech. Anti‐HEY1 (1:1000 dilution, Cat# ab154077, RRID:AB_2893447), anti‐OCT4 (1:1000 dilution, Cat# ab181557, RRID:AB_2687916), anti‐Nanog (1:1000 dilution, Cat# ab109250, RRID:AB_10863442), and anti‐SOX2 (1:1000 dilution, Cat# ab92494, RRID:AB_10585428) from Abcam. Anti‐Phosphoserine/threonine (1:1000 dilution, Cat# 612548, RRID:AB_399843) from BD Biosciences. Anti‐USP28 (1:1000 dilution, Cat# 4217, RRID:AB_1581796), anti‐HEY1 (1:1000 dilution, Cat# 19929‐1‐AP, RRID:AB_10646438), and anti‐Nanog (1:1000 dilution, Cat# 14295‐1‐AP, RRID:AB_1607719) from Proteintech. Anti‐phospho‐IKKα/β (ser176/180) (1:1000 dilution, Cat# bs‐3237R, RRID:AB_10883648) from Bioss. Anti‐Pd‐l1 (1:2000 dilution, Cat# HA722184) and anti‐PD‐L1 (1:2000 dilution, Cat# HA721176) from HUABIO. For detection, the following secondary antibodies from Thermo Fisher Scientific were diluted 1:5000 in 5% non‐fat milk: HRP‐conjugated Goat anti‐Mouse IgG (H+L) (Cat# 31430; RRID:AB_228307) and HRP‐conjugated Goat anti‐Rabbit IgG (H+L) (Cat# 32460; RRID:AB_1185567).

### 4.4 Immunoblotting (IB) and Immunoprecipitation (IP) Assays

IB and IP assays were performed as described previously [32]. Briefly, cellular proteins were extracted using IP lysis buffer (Thermo Fisher Scientific 87788) or RIPA buffer (Thermo Fisher Scientific 89901), each supplemented with protease inhibitors (Thermo Fisher Scientific A32965) and phosphatase inhibitor (Roche A32957). Protein concentrations were determined with the Bio‐Rad assay reagent. For western blot analysis, equal protein quantities were resolved by SDS‐PAGE and immunoblotted with indicated antibodies. For IP assay, cell lysates were incubated with specific primary antibodies overnight at 4°C, followed by a 3–4 h incubation with Protein A/G (Thermo Fisher Scientific 78610) or Protein G Sepharose beads (Thermo Fisher Scientific 88848). After extensive washing, the bead‐bound complexes were eluted, separated by SDS‐PAGE, and analyzed by immunoblotting. Immunoreactive bands were visualized with an enhanced chemiluminescence reagent (Thermo Fisher Scientific 34096).

### 4.5 In Vivo Ubiquitination Assays

Ubiquitination assays were conducted according to established protocols [19, 32]. In brief, cells transfected with the indicated constructs were cultured for 36–48 h. Prior to lysis with IP buffer, cells were treated with the proteasome inhibitor MG132 (20 µm) for 6–8 h. Lysates were subjected to immunoprecipitation using antibodies against the tagged proteins. Following three washes with IP buffer, the immunoprecipitated complexes were analyzed by SDS‐PAGE and immunoblotted with antibodies against the tags on ubiquitin.

### 4.6 Protein Half‐Life Assays

The half‐life of the HEY1 protein was determined using a CHX chase assay as previously described [19, 32]. Briefly, after transfection or treatment as specified, cells were exposed to CHX at a final concentration of 100 µg/mL to halt new protein synthesis. Cells were then collected at indicated time points, and the levels of the HEY1 protein were assessed by IB analysis.

### 4.7 Immunoprecipitation‐Mass Spectrometry (IP‐MS) Analysis of HEY1 Phosphorylation

After transfection HEK293T cells with HA‐HEY1 plus either empty vector (EV) or Flag‐IKKβ, cells were lysed and subjected to IP using an anti‐HA antibody. Immunoprecipitated HA‐HEY1 protein complexes were resolved by SDS‐PAGE on an 8% gradient gel and visualized via Coomassie blue staining. The HEY1 protein band was excised for subsequent analysis. Phosphopeptide identification was performed by tandem mass spectrometry (MS/MS) following in‐gel trypsin digestion. Acquired MS/MS spectra were processed using the Proteome Discoverer (PD) software (v.2.4), searching against the HEY1 protein sequence with Trypsin/P specified as the cleavage enzyme (allowing up to X missed cleavages). The search parameters included a precursor ion mass tolerance of 10 ppm (initial search) and a fragment ion mass tolerance of 0.02 Da; only peptides with scores >20 and confidence set to High were considered for identification.

### 4.8 In Vitro Kinase Assay

Flag‐HEY1 WT, Flag‐HEY1 S40A, or Flag‐HEY1 S40D were immunoprecipitated from HEK293T cells using Flag‐tag Protein IP Assay Kit with Agarose Gel (F2202M, Beyotime). HA‐IKKβ protein was purified by HA‐tag Protein IP Assay Kit with Magnetic Beads (F2185M, Beyotime). Flag‐HEY1 proteins were incubated with or without purified HA‐IKKβ in kinase buffer (Cat# 9802, Cell Signaling Technology) supplemented with 200 µm adenosine 5’‐triphosphate (Cat# 9804, Cell Signaling Technology) for 30 min at 30°C. The reaction was terminated by adding 10 mM EDTA. The samples were incubated with 5 µL of anti‐Flag antibody on a rotator at 4°C for 6 h. Protein complexes were then collected by adding 60 µL of Protein G Sepharose beads (Thermo Fisher Scientific, Cat# 88848) and incubating for another 6 h at 4°C. After washing the beads three times with 1 mL of pulldown buffer, the bound proteins were released by boiling in 2 × SDS‐PAGE sample buffer and resolved by SDS‐PAGE for immunoblot analysis with the designated antibody.

### 4.9 Real‐Time RT‐PCR Analysis

Total RNA isolation from both cellular and tissue samples was performed using TRIzol reagent (Invitrogen) following the manufacturer's protocol. For subsequent analysis, equal quantities of RNA were converted to cDNA with the TaqMan Reverse Transcription Reagents kit (Biosystems). Quantitative PCR was then conducted on a 7500 Real‐Time PCR System utilizing SYBR Select Master Mix (TakaRa, Cat# RR420A). The data shown in the figures represent mean ± SEM from at least three independent replicates. GAPDH expression was used to normalize RT‐PCR data across all samples. The primer sequences used are listed below: hHEY1, Forward: 5’‐ATCTGCTAAGCTAGAAAAAGCCG‐3’, Reverse: 5’‐GTGCGCGTCAAAGTAACCT‐3’; hGAPDH Forward: 5’‐TGGTATCGTGGAAGGACTC‐3’, Reverse: 5’‐AGTAGAGGCAGGGATGATG‐3’. mCasp1 Forward: 5’‐AATACAACCACTCGTACACGTC‐3’, Reverse: 5’‐AGCTCCAACCCTCGGAGAAA‐3’; mCcl20 Forward: 5’‐ACTGTTGCCTCTCGTACATACA‐3’, Reverse: 5’‐GAGGAGGTTCACAGCCCTTTT‐3’; mCxcl10 Forward: 5’‐CCAAGTGCTGCCGTCATTTTC‐3’, Reverse: 5’‐TCCCTATGGCCCTCATTCTCA‐3’; mStat1 Forward: 5’‐GCTGCCTATGATGTCTCGTTT‐3’, Reverse: 5’‐TGCTTTTCCGTATGTTGTGCT‐3’; mB2m Forward: 5’‐TGGTGCTTGTCTCACTGACC‐3’, Reverse: 5’‐TTCAGTATGTTCGGCTTCCC‐3’; mH2‐K1 Forward: 5’‐GCTGGTGAAGCAGAGAGACTCAG‐3’, Reverse: 5’‐GGTGACTTTATCTTCAGGTCTGCT‐3’; mH2‐D1 Forward: 5’‐AGTGGTGCTGCAGAGCATTACAA‐3’, Reverse: 5’‐GGTGACTTCACCTTTAGATCTGGG‐3’; mPsmb8 Forward: 5’‐GTGCAGGTTGTATTATCTTCGGA‐3’, Reverse: 5’‐CGAGTCCCATTGTCATCTACG‐3’; mPsmb7 Forward: 5’‐GTGTCGGTGTTTCAGCCAC‐3’, Reverse: 5’‐GTGCCAGTTTTCCGAGCTTTC‐3’; mPsmb6 Forward: 5’‐5‐GGACAACCACTGGGTCCTAC‐3’, Reverse: 5’‐CAAGCTGGTAAGTGACAGCGT‐3’; mTapbp Forward: 5’‐GGCCTGTCTAAGAAACCTGCC‐3’, Reverse: 5’‐CCACCTTGAAGTATAGCTTTGGG‐3’;

mErap1 Forward: 5’‐TAATGGAGACTCATTCCCTTGGA‐3’, Reverse: 5’‐AAAGTCAGAGTGCTGAGGTTTG‐3’; mGAPDH Forward: 5’‐AATGGATTTGGACGCATTGGT‐3’, Reverse: 5’‐TTTGCACTGGTACGTGTTGAT‐3’.

### 4.10 RNA Extraction and Sequencing Analysis

Total RNA was isolated from shUsp28‐ or NT shRNA‐infected Hepa1‐6 cells using a Trizol‐based kit (B511311, Sangon Biotech, China) and treated with RNase‐free DNase I. Qualified RNA was transferred to Sangon Biotech (Shanghai, China) for sequencing services. Libraries for RNA sequencing were prepared from 1 µg of input RNA using the VAHTSTM mRNA‐seq V2 Kit designed for the Illumina platform.

Data processing included read alignment to the GRCm39 genome (STAR v2.7.10a) [48], transcript quantification (Salmon v1.9.0) [49] to generate TPM‐normalized profiles and raw counts, and differential expression analysis with DESeq2 [50] (significance cutoffs: |log_2_FC|> 1, *p* < 0.05). The overall methodology has been described previously [32].

For functional annotation, Gene Ontology (GO) enrichment, Gene Set Enrichment Analysis (GSEA), and Kyoto Encyclopedia of Genes and Genomes (KEGG) pathway analysis were conducted using the clusterProfiler R package (v4.16.0) [51] based on differentially expressed genes (DEGs). All pathway annotations were downloaded from the MSigDB database (GSEA | MSigDB | Browse Mouse Gene Sets). For visualization, ggplot2 (v3.5.2) and pheatmap (v1.0.12) were used to generate volcano plots, box plots, and heatmaps for DEG clustering.

### 4.11 Data Collection

Transcriptomic profiles and associated clinical data for HCC patients were acquired from the TCGA‐LIHC cohort (n = 370) via the UCSC XENA (https://xenabrowser.net/). GSE210287 (*n* = 22) [52], GSE93157 (*n* = 65) [53], GSE135222 (*n* = 22) [54], GSE207422 (*n* = 39) [55], GSE67501 (*n* = 11) [56], and GSE89377 (*n* = 107) [57] were derived from the Gene Expression Omnibus (GEO) database (GEO Accession viewer). IMvigor210 (*n* = 90) was from R package IMvigor210CoreBiologies (Index of /IMvigor210CoreBiologies/packageVersions/).

### 4.12 Flow Cytometry

Cells were washed once with PBS and then surface‐stained with PE‐conjugated anti‐PD‐L1 or APC‐conjugated anti‐H2K^d^/H2D^d^ antibodies in PBS for 30 min at 4°C. After staining, cells were fixed and permeabilized using the eBioscience kit under the same conditions. Following a PBS wash, flow cytometric analysis was performed on a Beckman CytoFLEX instrument, and data were acquired with CytExpert Software 2.3. Final data processing and analysis were conducted using FlowJo.

### 4.13 Immunofluorescence (IF) Analysis

IF staining was performed as previously reported [32]. Briefly, cultured cells were fixed with 4% paraformaldehyde (PFA), permeabilized with 0.1% Triton X‐100 for 5 min, and blocked with 10% FBS for 1 h. Cells were then immunolabeled by incubation with primary antibodies overnight at 4°C. Subsequently, appropriate Alexa Fluor‐conjugated secondary antibodies (goat anti‐rabbit IgG‐AF647, A32733; goat anti‐mouse IgG‐AF488, A11001; Life Technologies/Molecular Probes) were applied along with DAPI (Sigma–Aldrich, D9542) for nuclear counterstaining. Fluorescent images were acquired using a Leica confocal microscope.

### 4.14 Immunohistochemical (IHC) Staining

IHC was conducted in accordance with established protocols [19, 28]. Formalin‐fixed, paraffin‐embedded human HCC tissue microarrays (HLivH180Su16; Shanghai OUTDO Biotech; Approval SHYJS‐CP‐1707005) were utilized. Staining was automated on a DAKO Autostainer using the LSAB+ detection system with diaminobenzidine (DAB) chromogen. Primary antibodies and their dilutions included: anti‐HEY1 (ab154077, 1:200), anti‐USP28 (ab244290, 1:100), anti‐CD8 (85336, 1:200), anti‐TIM‐3 (83882, 1:100), anti‐PD‐L1 (1:400), and anti‐GzmB (1:200). The final IHC score for each tissue section was calculated by multiplying the staining intensity score by the percentage score of positive cells, as described previously [28]. Briefly, the percentage of positive tumor cells (extent score: 0–4) was multiplied by the staining intensity (intensity score: 0–3) to generate a final expression score ranging from 0 to 12. The percentage categories were defined as: 1 (1%–25%), 2 (26%–50%), 3 (51%–75%), and 4 (76%–100%). Intensity was graded as: 1 (weak), 2 (moderate), and 3 (strong).

### 4.15 Colony and Sphere Formation Assays

Colony and sphere formation assays were performed as described [6, 19]. For sphere formation, cells were seeded at a density of 10 cells per well in ultra‐low attachment 96‐well plates (Corning) and cultured in DMEM/F12 medium (Gibco) containing B27 supplement (Gibco), 20 ng/mL epidermal growth factor (Peprotech), 20 ng/mL basic fibroblast growth factor (Peprotech), and 10 ng/mL hepatocyte growth factor (Peprotech). To minimize cell aggregation, 1% methylcellulose was included in the medium. For colony formation, tumor cell lines (PLC/PRF/5, Huh7, and Hepa1‐6) were plated in 24‐well plates at densities of 200 or 500 cells per well and cultured for 10–16 days. Colonies were washed with PBS, fixed with 10% acetic acid/10% methanol for 30 min, and stained with 0.4% crystal violet in 20% ethanol. After rinsing with distilled water and air‐drying, colonies were quantified and analyzed.

### 4.16 Ex Vitro T Cell co‐culture Assay

The ex vitro T cell co‐culture assay was performed as previously detailed [19]. Briefly, CD8^+^ T cells were purified from murine splenocyte suspensions via magnetic‐activated cell sorting using the Mouse CD8 (Ly‐2) MicroBeads kit (Miltenyi Biotec, Cat# 130‐117‐044). Cells were subsequently stimulated for 48 h with mouse anti‐CD3/CD28 antibodies (Gibco, Cat# 11453D) in the presence of human IL‐2 (50 U/mL; Novoprotein, Cat# CK24). Activated T cells were then co‐cultured with target tumor cells at an effector‐to‐target ratio of 10:1. For functional readouts, co‐cultures were set up in different formats: (i) In 96‐well plates for 24 h to measure caspase‐3/7 activity using the Caspase‐Glo 3/7 Assay (Promega, Cat# G8092); (ii) In 24‐well plates for 48 h in the presence or absence of AZ‐1, followed by crystal violet staining of adherent tumor cells; and (iii) In 24‐well plates for 24 h to quantify tumor cell death using the Live‐Dead Cell Imaging Kit (Invitrogen, Cat# R37601). All assays were conducted according to the manufacturers’ guidelines.

### 4.17 Mouse Xenograft Assays

All animal experiments complied with relevant ethical regulations and were approved by the Ethics Committee of the Second Affiliated Hospital of Chongqing Medical University (Approval IACUC‐SAHCQMU‐2025‐0137). Mice were randomly distributed into groups. As previously described [19], Hepa1‐6 (2.5‐5.0 × 10^6^), H22 (2.5 × 10^6^), or PLC/PRF/5 (1.0 × 10^6^) cells were suspended in 100 µL DMEM, mixed 1:1 with Matrigel (Corning, 354234), and injected subcutaneously into both flanks of 5‐7‐week‐old male C57BL/6 or 4‐6‐week‐old male NOD/SCID mice. Tumor growth was monitored by caliper measurement every three days, with volume calculated as (L × W^2^ × *π*/6). Once tumors reached the ethical size limit (< 15 mm), mice were euthanized, tumors were dissected, and their weights were documented.

### 4.18 In Vivo Experimental Therapy in Mouse Model

H22 or Hepa1‐6 cells were injected into 5–6 week‐old male C57BL/6 mice to establish subcutaneous tumors. For depletion experiments, mice received intraperitoneal injections of 200 µg of anti‐CD8 (clone 2.43, Bio X Cell), anti‐CD4 (clone GK1.5, Bio X Cell), or anti‐NK1.1 (clone PK136, Bio X Cell) antibodies on days 0 and 7. In therapeutic experiments using the H22 and Hepa1‐6 models, mice were randomly assigned to four treatment groups: (i) isotype control antibody (Biolegend, RTK4530), (ii) anti‐PD‐1 monoclonal antibody (Bio X Cell, clone RMP1‐14), (iii) the USP28 inhibitor AZ‐1, and (iv) a combination of AZ‐1 and anti‐PD‐1. For the Hepa1‐6 model with Usp28 knockdown, mice were allocated to either control antibody or anti‐PD‐1 treatment groups. Control and anti‐PD‐1 antibodies were administered intraperitoneally at 100 µg per mouse in 88 µL HBSS saline buffer. AZ‐1 was delivered via intraperitoneal injection at a dose of 25 mg/kg, formulated in a vehicle of DMSO/PEG300 (1:1, 100 µL volume). Treatments were administered every two days for a total of seven doses. In survival studies, mice were monitored until tumors reached a volume exceeding 1500 mm^3^ or developed ulceration with a diameter ≥ 15 mm. All statistical analyses were performed using GraphPad Prism software.

### 4.19 Statistical Analysis

All statistical analyses were conducted using GraphPad Prism software (version 10). Quantitative data from western blotting were obtained by densitometric analysis of the original blot images with ImageJ software. Data are presented as mean ± standard error of the mean (S.E.M.) derived from a minimum of three independent biological replicates. Group comparisons for tumor volume and other quantitative results were performed using the appropriate statistical tests, including two‐tailed unpaired or paired Student's *t*‐test, or two‐way ANOVA. Statistical significance was defined as *p* < 0.05 (^*^ *p* < 0.05; ^**^ *p* < 0.01; ^***^ *p* < 0.001). Survival analysis were assessed using Kaplan‐Meier curves, and differences between groups were evaluated by the log‐rank test.

## Author Contributions

C. L. directed the project, designed and wrote the manuscript; N. S., L. Z., and Q. Z. performed the experiments; G. S. performed IHC staining of human and mouse tumor samples; Y. L. and Y. C. collected human liver cancer tissues; T. F. performed bioinformatic analysis; N. S. and C. L. conducted data analysis; F. X. edited the manuscript. All authors commented on the manuscript.

## Conflicts of Interest

The authors declare no conflicts of interest.

## Supporting information




**Supporting File 1**: advs75843‐sup‐0001‐SuppMat.docx.

**Supporting File 2**: advs75843‐sup‐0002‐FigureS1‐S9.zip.

## Data Availability

All reagents employed in this study are commercially sourced or available from the corresponding author upon reasonable request. The RNA sequencing data generated in this work are accessible in the Gene Expression Omnibus (GEO) database under accession number GSE389752. The proteomics dataset has been submitted to the ProteomeXchange Consortium via the PRIDE repository with the identifier PXD065981. Source data for the figures are included with this article. Any additional data supporting the conclusions of this study are available from the corresponding author upon reasonable request.
