# Supplementary material for: IKKβ and USP28 Regulate HEY1 Stability to Promote Cancer Stemness and Immune Evasion in Hepatocellular Carcinoma
Source: Adv Sci (Weinh). 2026 May 26:e75843. Online ahead of print. doi: 10.1002/advs.75843 (PMC13335928; doi:10.1002/advs.75843)
Supplement: Supplementary file 1 — Supporting File 1: advs75843‐sup‐0001‐SuppMat.docx. [file ADVS-9999-e75843-s001.docx]

**IKKβ and USP28 regulate HEY1 stability to promote cancer stemness and immune evasion in hepatocellular carcinoma**

Na Shao^1^, Lin Zhang^2^, Gufang Shen^2^, Yangfan Lv^1^, Tianshu Fang^2^, Ya Cao^1^, Qiongyi Zhang^3^, Feng Xu^4^, Chungang Liu^2*^

^1^Department of Pathology, Xinqiao Hospital, Third Military Medical University, Chongqing, 400010, PR China.

^2^Key Laboratory of Molecular Biology for Infectious Diseases (Ministry of Education), Institute for Viral Hepatitis, Department of Infectious Diseases, The Second Affiliated Hospital, Chongqing Medical University, Chongqing 400010, PR China.

^3^Institute of Molecular and Cell Biology, Agency for Science, Technology and Research (A*STAR), Singapore 138673, Republic of Singapore.

^4^Hengrui-Singapore Innovation Centre for Chronic Diseases, Singapore 138673, Republic of Singapore.

*Correspondence: C. L.: [liuchungang@zju.edu.cn](mailto:liuchungang@zju.edu.cn).

**
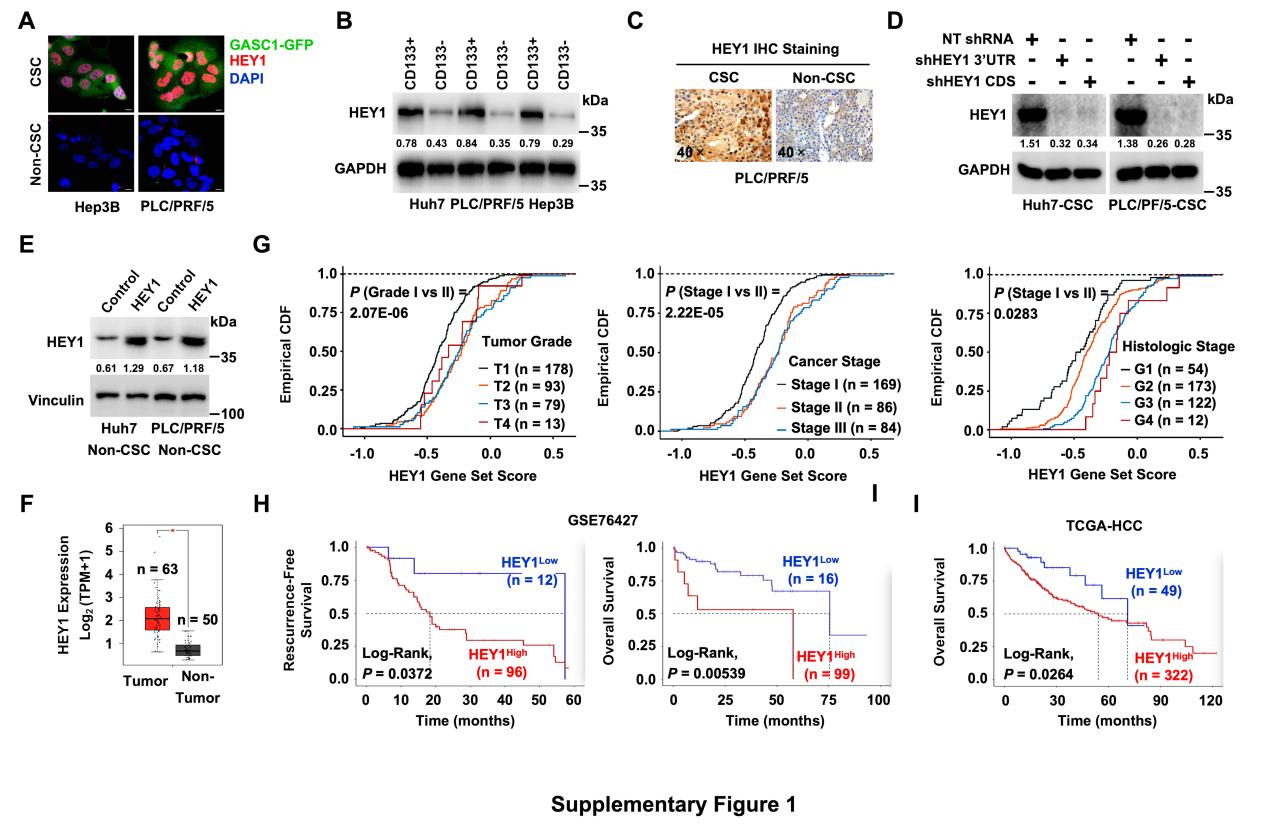
**

**
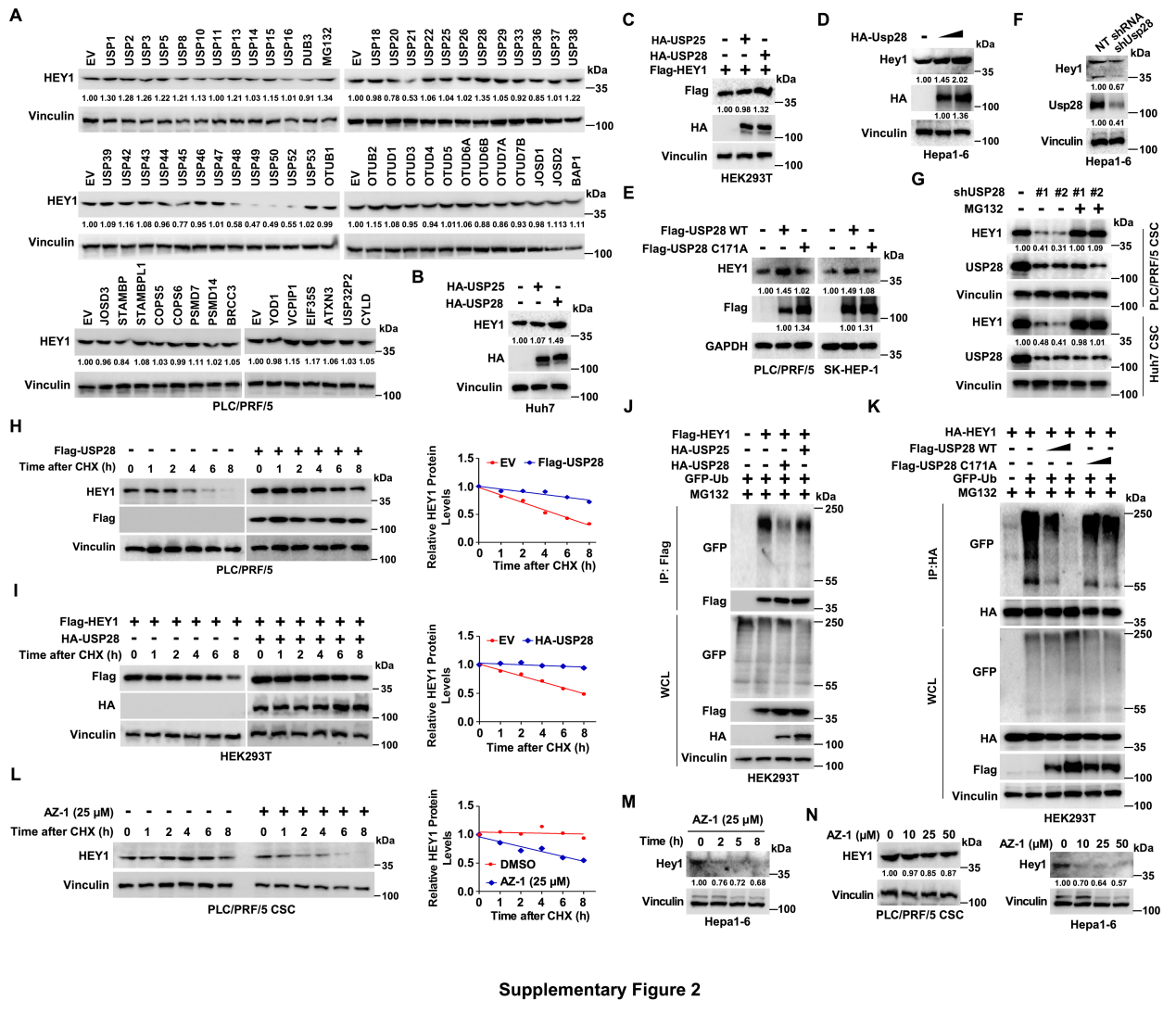
**

**
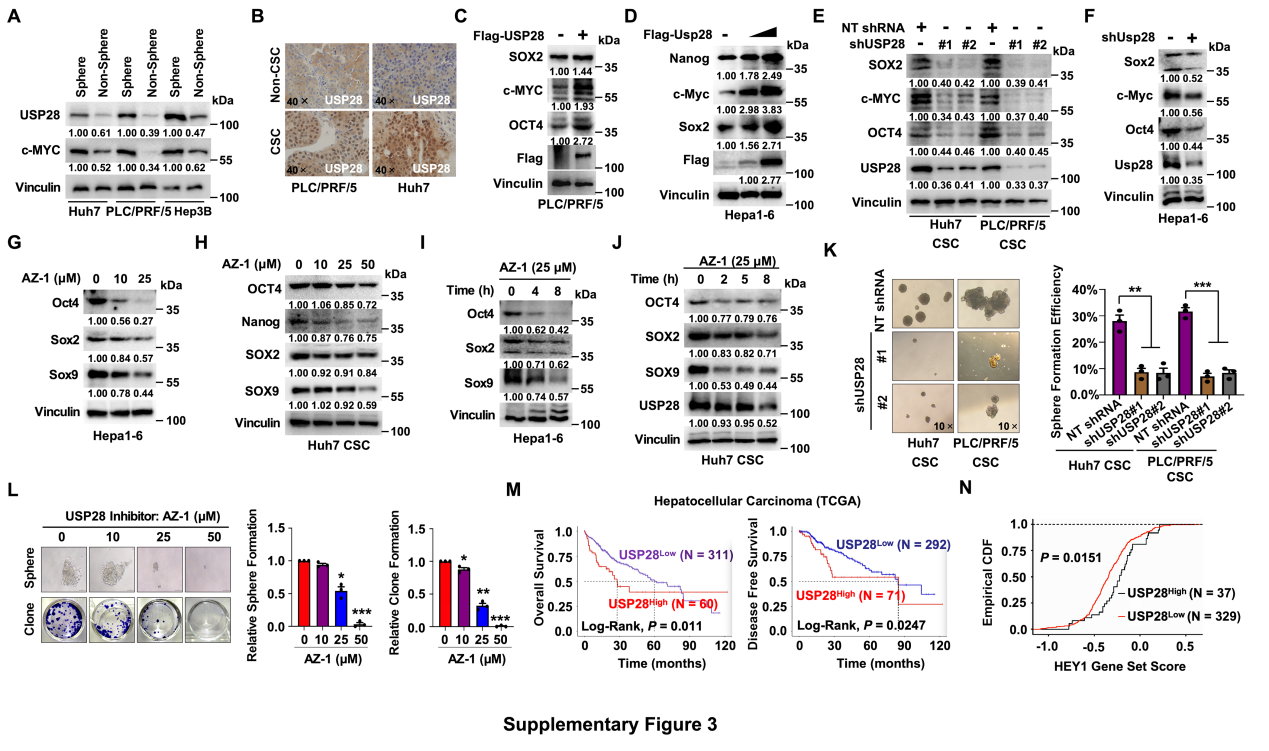
**

**
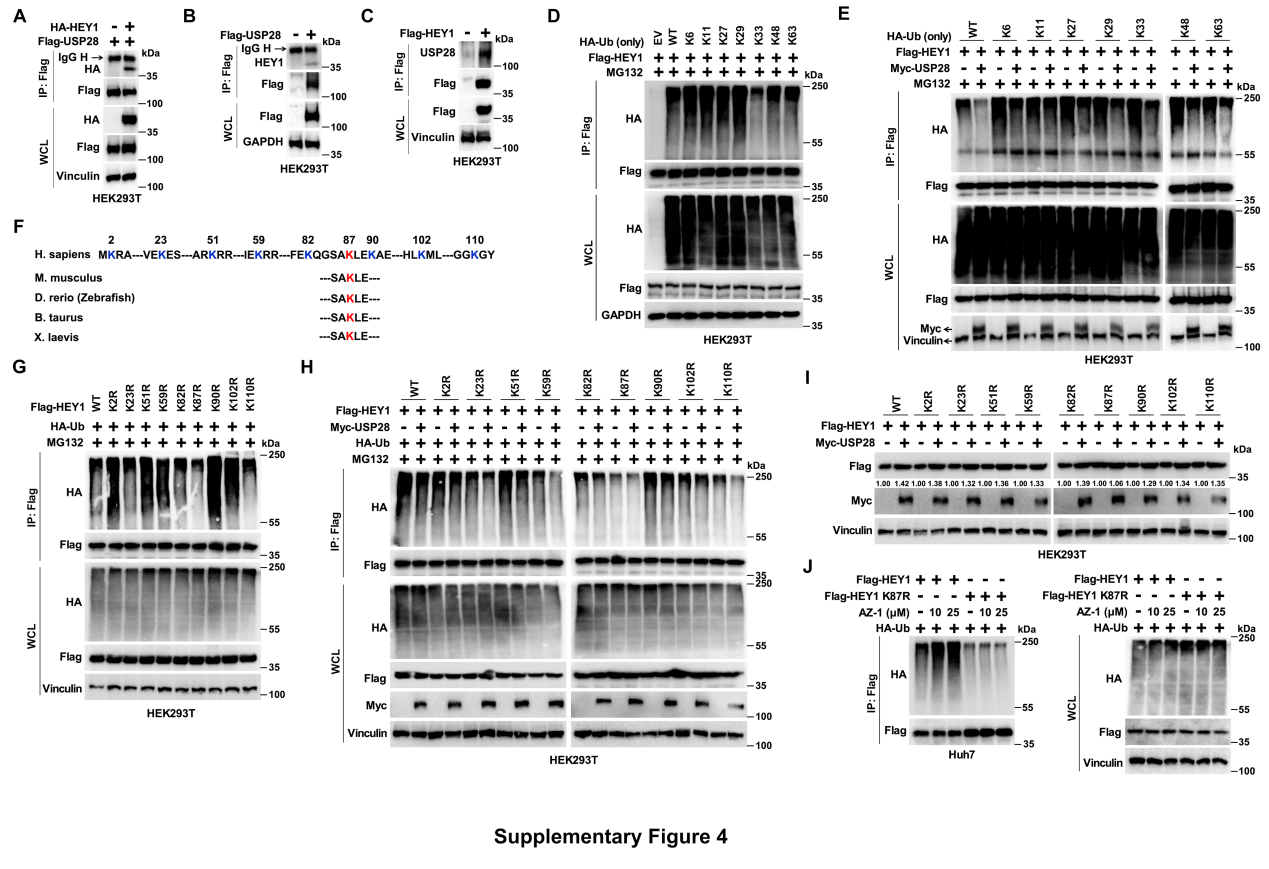
**

**
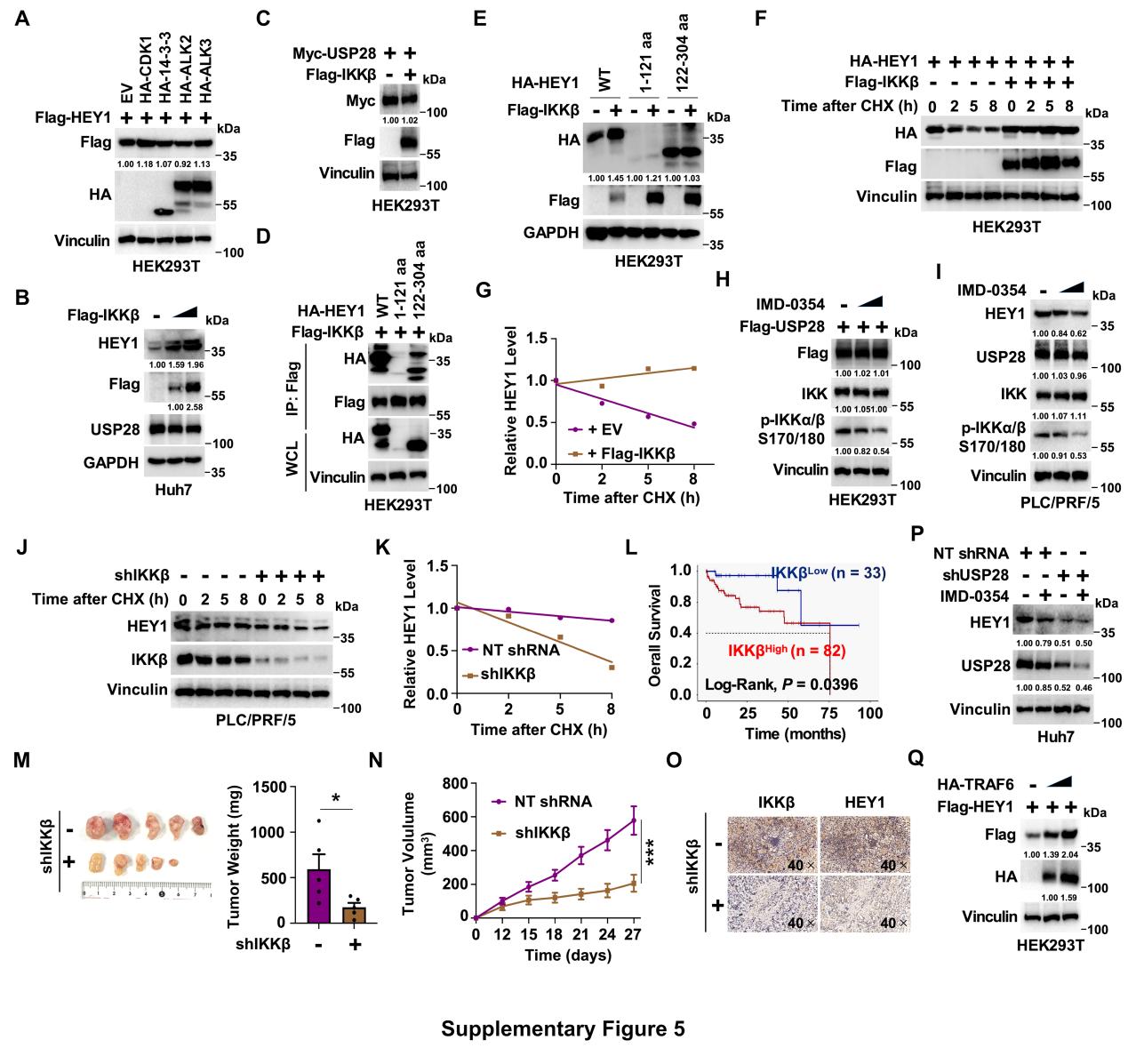
**

**
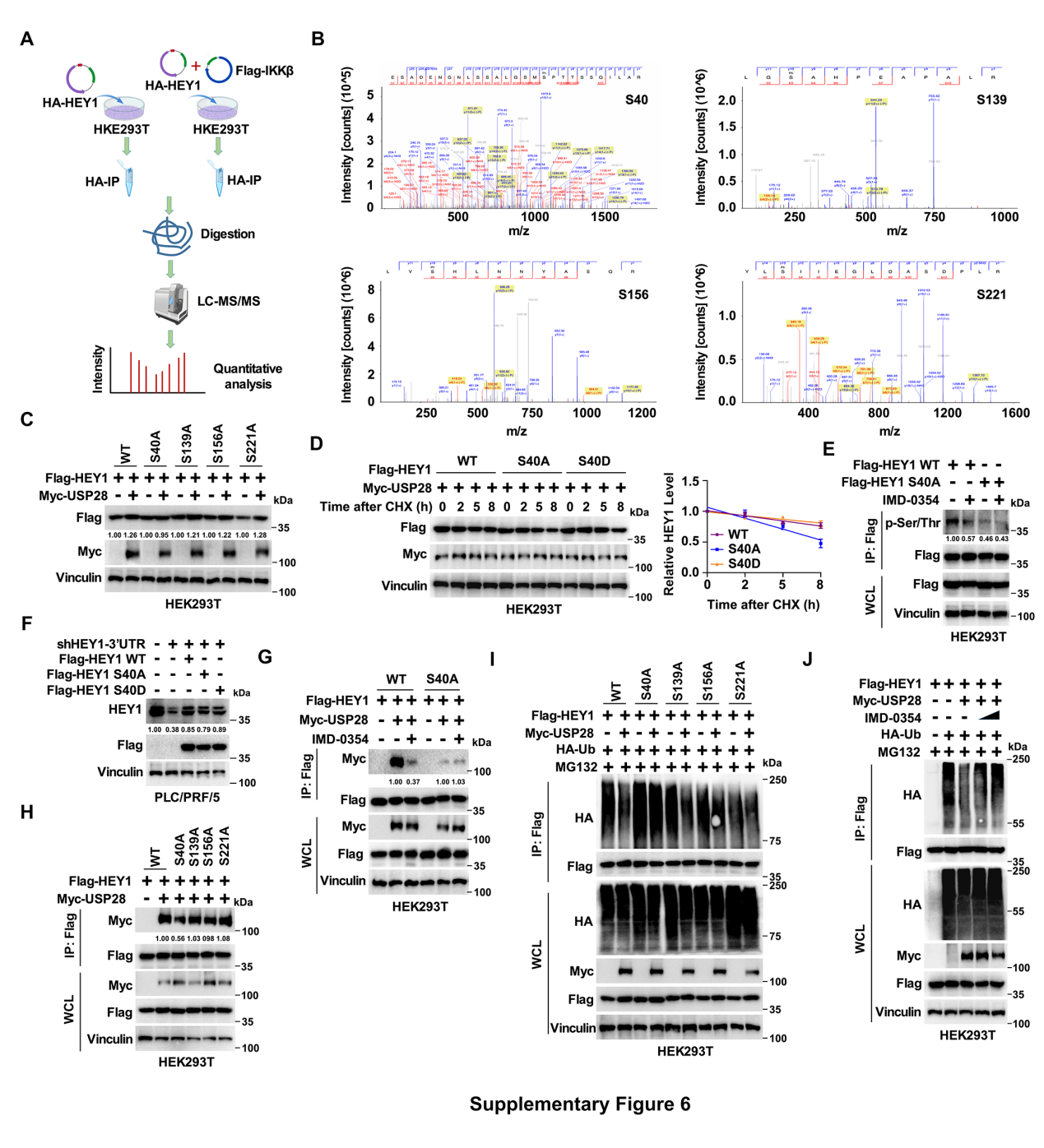
**

**
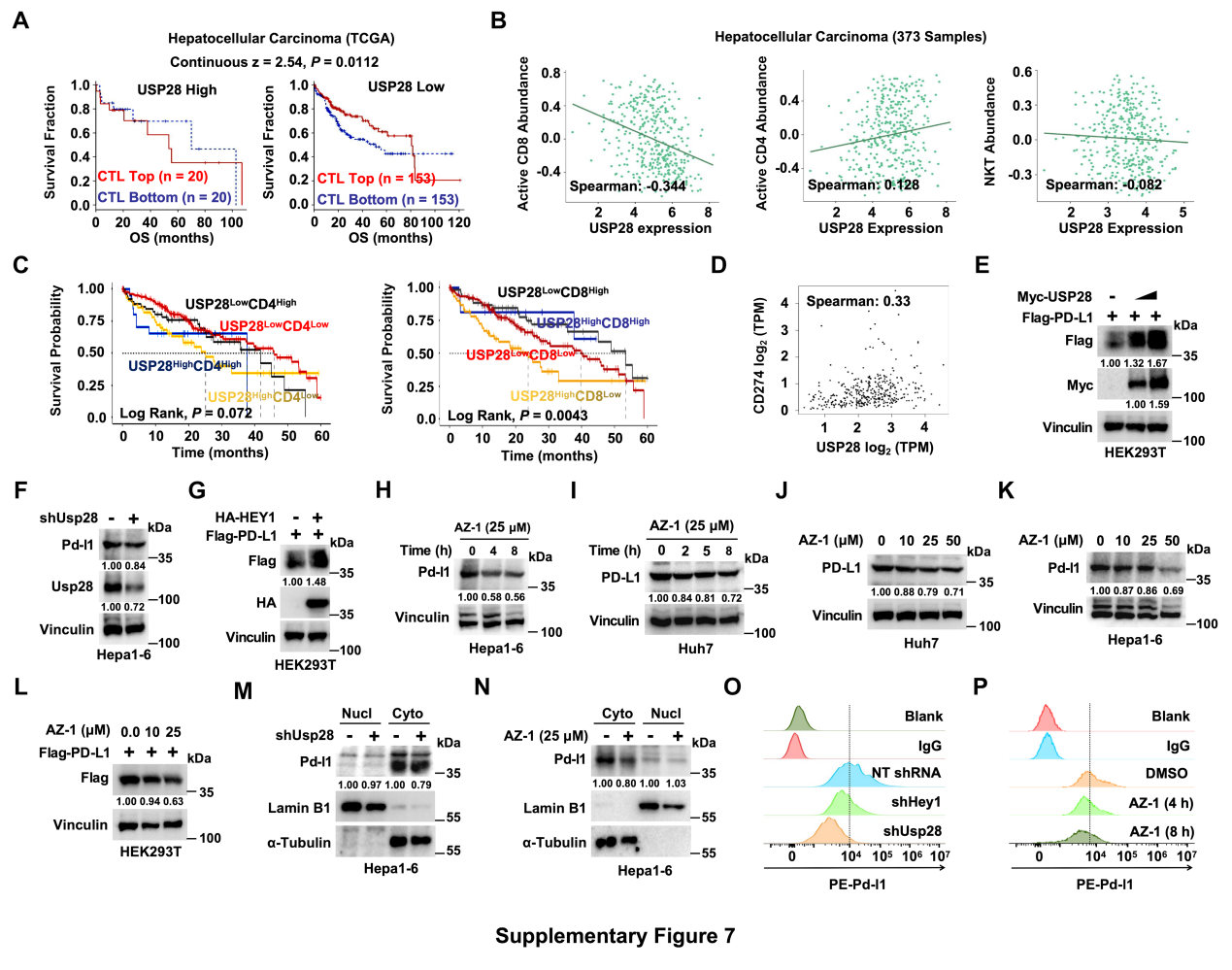
**

**
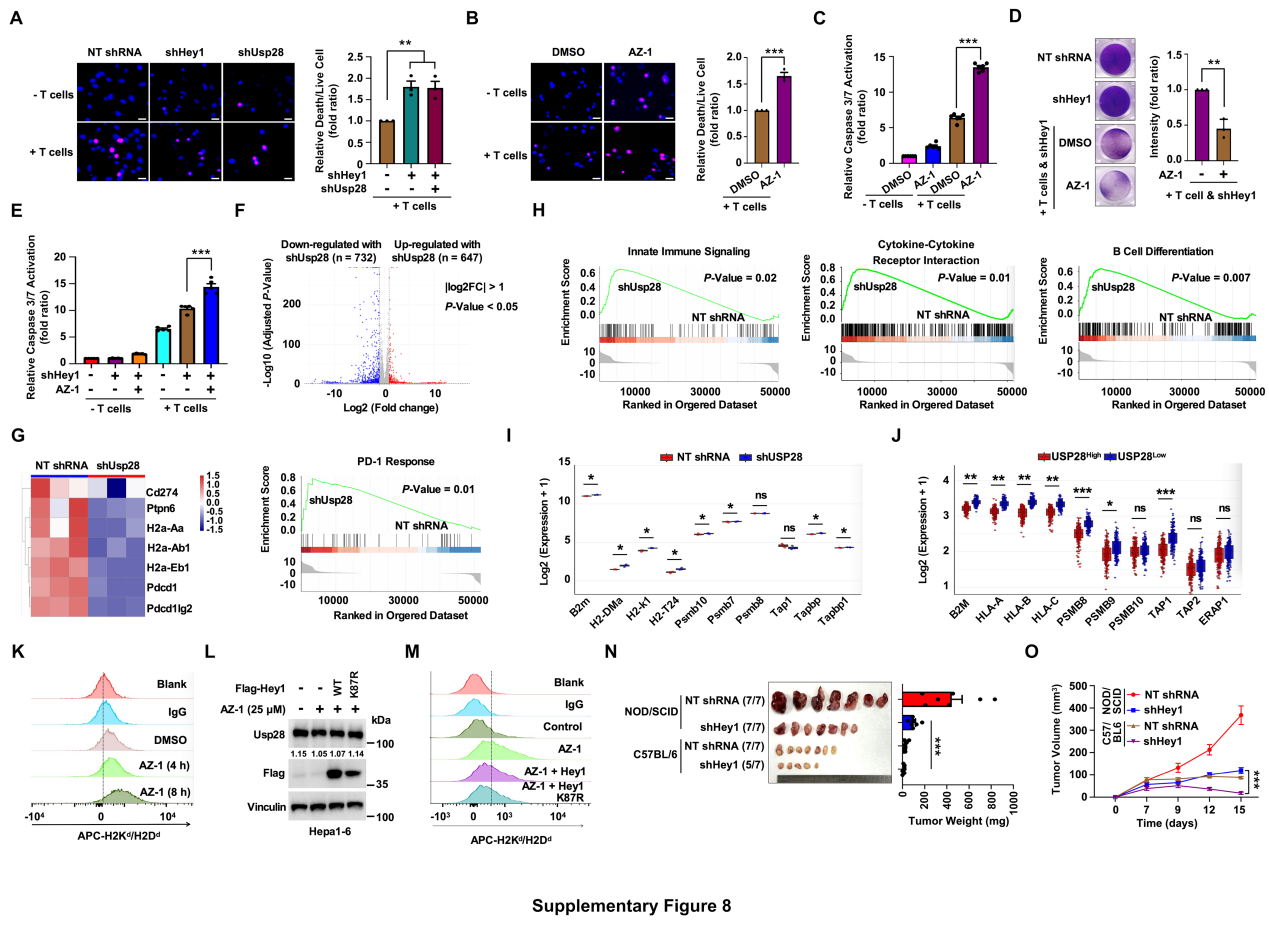
**

**
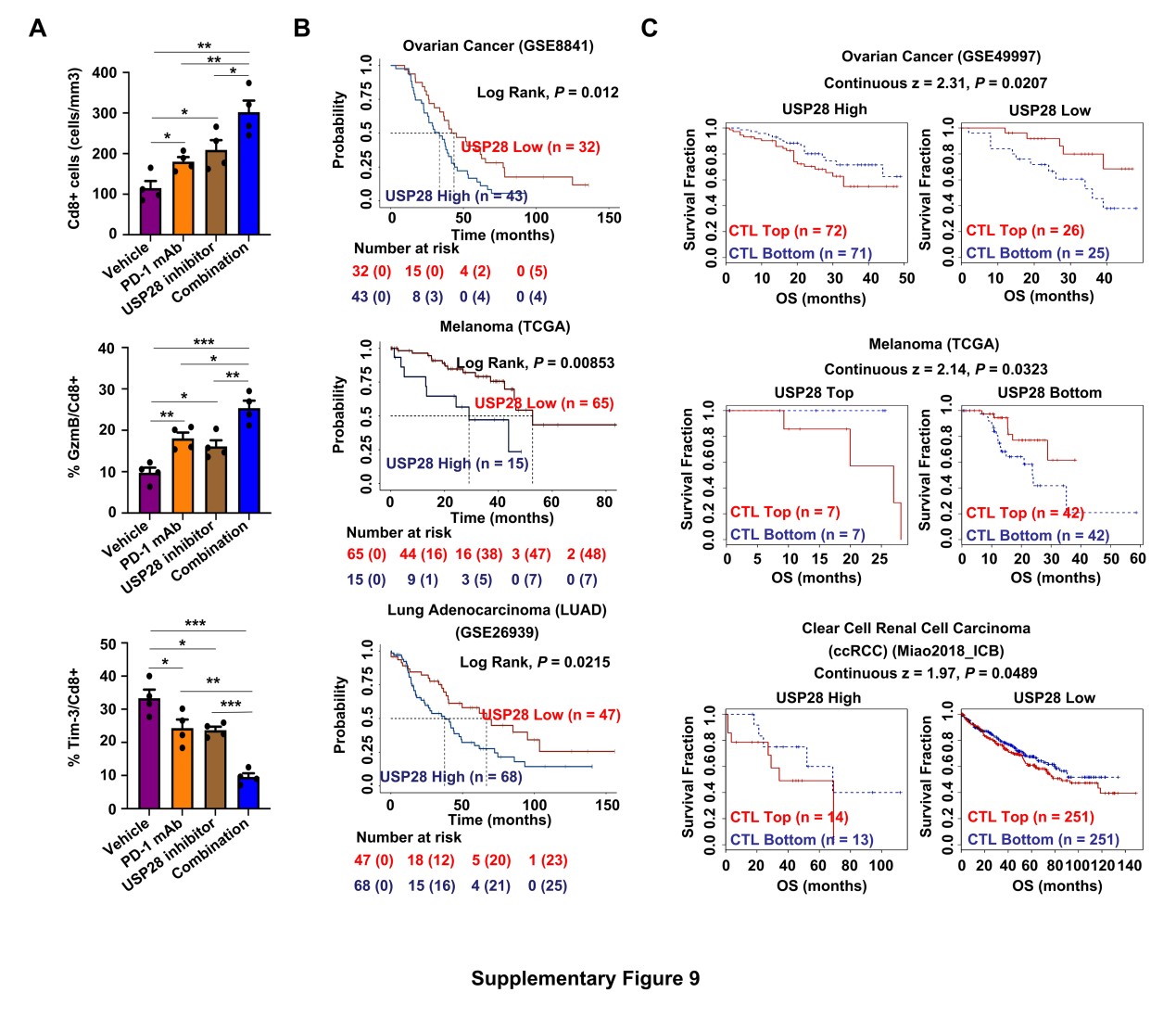
**

**Supplementary Figure Legends**

**Supplementary Fig 1. HEY1 is highly expressed in liver CSCs and correlates with poor prognosis in HCC patients.**

**A, B** Immunofluorescence (IF) and immunoblot (IB) analysis of HEY1 expression in liver CSC and non-CSC populations purified from different HCC cell lines by FACS. Scale bars, 20 μm.

**C** Immunohistochemical (IHC) staining for HEY1 expression in liver CSC and non-CSC tumors derived from PLC/PRF/5 subcutaneous xenografts. Representative images are shown. Scale bars, 5 μm.

**D** IB analysis of WCL from Huh7 and PLC/PRF/5 CSC cells with HEY1 knockdown by shRNA.

**E** IB analysis of WCL from Huh7 and PLC/PRF/5 non-CSC cells stably expression of HEY1.

**F** HEY1 expression in liver tumor and non-tumor tissues from the GEPIA2 database.

**G** Empirical cumulative distribution function (CDF) plots showing correlation of individual tumors with HEY1 signature across various tumor grades, clinical stages and histologic stages within the HCC cohort.

**H, I** Kaplan-Meier analysis of HEY1 expression and overall survival of HCC patients.

**Supplementary Fig 2. USP28 positively regulates HEY1 protein stability.**

**A** IB analysis of endogenous HEY1 protein levels in PLC/PRF/5 cells transfected with indicated constructs encoding various DUB proteins. Cells were treated with 20 µM MG132 for 8 h as a positive control.

**B, C** IB analysis of HEY1 protein levels in Huh7 (**B**) or HEK293T (**C**) cells expressing the indicated constructs.

**D** IB analysis of Hey1 protein levels in Hepa1-6 cells expressing increasing amounts of HA-Usp28.

**E** IB analysis of HEY1 protein levels in PLC/PRF/5 or SK-HEP-1 cells expressing Flag-USP28 or Flag-USP28 C171A.

**F** IB analysis of Hey1 protein levels in USP28-KD Hepa1-6 cells.

**G** IB analysis of WCL derived from PLC/PRF/5 or Huh7 CSC cells with USP28 knockdown. Cells were treated with or without 20 μM MG132 for 8 h before harvesting.

**H, I** Protein half-life assay was performed for the assessment of HEY1 stability in PLC/PRF/5 (**H**) or HEK293T (**I**) cells with USP28 overexpression. 36 h after transfection, cells were treated with 100 µg/mL CHX for the indicated time period before they were harvested for IB analyses. Quantification of HEY1 levels relative to Vinculin was shown (Right Panel).

**J** In *vivo* ubiquitination analysis of HEY1 in HEK293T cells overexpressing HA-USP25 or HA-USP28 plasmids. A total of 36 h after transfection, cells were treated with 20 μM MG132 for 8 h before they were harvested. Flag-HEY1 was immunoprecipitated with anti-Flag antibody and immunoblotted with anti-GFP antibody which specifically recognizes GFP-tagged ubiquitin.

**K** In *vivo* ubiquitination analysis of HEY1 in HEK293T cells expressing increasing amounts of Flag-USP28 WT or Flag-USP28 C171A. A total of 36 h after transfection, cells were treated with 20 μM MG132 for 8 h before they were harvested.

**L** Protein half-life analysis of HEY1 in PLC/PRF/5 CSC cells treated with or without 25 μM AZ-1 for 24 h before performing the assay. Quantification of HEY1 levels relative to Vinculin was shown.

**M, N** IB analysis of HEY1 protein levels derived from PLC/PRF/5 CSC or Hepa1-6 cells treated with either 25 μM AZ-1 for varying time points (**M**), or different doses of AZ-1 for 24 h (**N**).

**Supplementary Fig 3. USP28 is enriched in liver CSCs and is essential for CSC self-renewal.**

**A** IB analysis of USP28 in oncosphere and non-oncosphere cells derived from various HCC cell lines.

**B** IHC staining for USP28 expression in liver CSC and non-CSC tumors derived from PLC/PRF/5 and Huh7 subcutaneous xenografts.

**C** IB analysis of indicted proteins in PLC/PRF/5 cells expressing Flag-USP28.

**D** IB analysis of indicted proteins in Hepa1-6 cells expressing increasing amounts of Flag-Usp28.

**E, F** IB analysis of indicated protein levels in different cancer cells indicated lentiviral shRNAs targeting USP28.

**G-J** IB analysis of indicated protein levels derived from different cancer cells treated with either different doses of AZ-1 for 24 h (**G, H**), or 25 μM AZ-1 for varying time points (**I, J**).

**K, L** Sphere and colony formation efficiency assays of liver CSCs with USP28 knockdown or treated with different doses of AZ-1. Data are presented as mean ± SEM. * *P* < 0.05, ** *P* < 0.01, *** *P* < 0.001. *t*-test.

**M** Kaplan-Meier analysis of USP28 expression and overall survival or disease-free survival of HCC patients.

**N** CDF plots showing correlation of individual tumors with HEY1 signature across USP28 expression levels within the HCC cohort.

**Supplementary Fig 4. USP28 deubiquitinates HEY1 at lysine 87.**

**A-C** IB analysis of WCL and Flag immunoprecipitate from HEK293T cells transfected with Flag-USP28 and HA-HEY1 (**A**), or Flag-USP28 alone (**B**) or Flag-HEY1 alone (**C**).

**D** HEY1 polyubiquitination linkage was examined by transfecting HA-tagged WT or indicated ubiquitin mutants containing Lys 6/11/27/29/33/48/63-only mutations (the other six of seven lysine residues were mutated to arginine) together with Flag-HEY1 into HEK293T cells, followed by IB analysis of HA-Ub in anti-Flag IP products. Cells were treated with 20 μM MG132 for 8 h before harvesting.

**E** Effects of USP28 overexpression on HEY1 polyubiquitination in HEK293T cells transfected with the indicated ubiquitin mutant plasmids. A total of 36 h after transfection, cells were treated with 20 μM MG132 for 8 h before they were harvested for Flag-tag IP and HA-Ub IB analyses.

**F** Conservation of the K87 patch among different species.

**G** In *vivo* ubiquitination assay of HEY1 in HEK293T cells expressing Flag-tagged WT-HEY1 or the indicated HEY1 K to R mutants in the presence of ectopic HA-Ub expression. After 36 h post transfection, cells were treated with 20 μM MG132 for 8 h before harvesting.

**H** Effects of USP28 overexpression on HEY1 polyubiquitination in HEK293T cells transfected with the indicated constructs. cells were treated with 20 μM MG132 for 8 h before they were harvested for Flag-tag IP and HA-Ub IB analyses.

**I** IB analysis of the protein levels of WT-HEY1 and the indicated HEY1 K to R mutants in HEK293T cells with or without ectopic Myc-USP28 expression.

**J** Effects of AZ-1 on HEY1 polyubiquitination. Huh7 cells expressing indicated plasmids were treated with different doses of AZ-1. The ubiquitinated HEY1 was immunoprecipitated by anti-Flag and analyzed by IB.

**Supplementary Fig 5. IKKβ signalling regulates HEY1 protein stability.**

**A** IB analysis of WCL derived from HEK293T cells co-transfected with Flag-HEY1 and the indicated HA-tagged kinases.

**B** IB analysis of HEY1 protein levels in Huh7 cells expressing increasing amount of Flag-IKKβ.

**C** IB analysis of USP28 protein levels in HEK293T cells expressing Flag-IKKβ. Cells were harvested 48 h after transfection with indicated plasmids.

**D** IB and IP products analysis of HEY1-IKKβ interaction in HEK293T cells expressing Flag-tagged WT IKKβ or the indicated truncated HEY1 mutants.

**E** IB analysis of the protein levels of the indicated truncated HEY1 mutants in HEK293T cells expressing Flag-IKKβ.

**F, G** IB analysis of WCL derived from HEK293T cells transfected with indicated constructs. 36 h post transfection, cells were treated with 100 μg/mL CHX at indicated time points (**F**). The HEY1 protein abundance was quantified by the ImageJ software and plotted (**G**).

**H, I** IB analysis using the indicated antibodies in HEK293T (**H**) and PLC/PRF/5 (**I**) cells treated with increasing concentrations of IMD-0354 for 24 h before harvesting.

**J, K** Protein half-life assay was performed for the assessment of HEY1 stability in PLC/PRF/5 cells with IKKβ knockdown by shRNA. Cells were treated with 100 μg/mL CHX for the indicated time period before they were harvested for IB analyses (**J**). Quantification of HEY1 levels relative to Vinculin was shown (**K**).

**L** Kaplan-Meier analysis of IKKβ expression and overall survival of HCC patients.

**M, N** In *vivo* tumor growth was measured at the indicated time points and tumors were dissected at the endpoint. Data are presented as mean ± SEM, n = 5 per group. * *P* < 0.05, *** *P* < 0.001.

**O** Loss of IKKβ leads to decreased HEY1 protein levels in the xenograft tumors. IHC analysis of lysates derived from dissected xenografts formed by IKKβ-depleted PLC/PRF/5 CSC cells.

**P** The WT and USP28-KD Huh7 cells were treated with 10 μM IMD-0354 for 36 h, then collected and analyzed by IB.

**Q** IB analysis of HEY1 protein levels in HEK293T cells expressing increasing amount of HA-TRAF6.

**Supplementary Fig 6.** IKKβ**-mediated phosphorylation of HEY1 at Ser40 promotes USP28 interaction.**

**A** Schematic illustration of the quantitative phosphoproteomics screening strategy for identifying HEY1 phosphorylation sites induced by ectopic IKKβ expression.

**B** HEY1 immunoprecipitated from HEK293T were separated by SDS-PAGE, and collected for mass spectrometry analysis. Mass spectrometry analysis revealed phosphorylation at serine residues S40, S139, S156, S221 in HEY1 after ectopic expression of IKKβ.

**C** IB analysis of WCL derived from HEK293T cells transfected with indicated constructs.

**D** IB analysis of WCL derived from HEK293T cells transfected with indicated constructs. 36 h post transfection, cells were treated with 100 μg/mL CHX at indicated time points (Left Panel). The HEY1 protein abundance was quantified by the ImageJ software and plotted (Right Panel).

**E** In *vivo* phosphorylation assay of Flag-tagged HEY1 constructs in HEK293T cells treated with 10 μM IMD-0354 for 24 h before harvesting.

**F** IB analysis of HEY1 protein levels in HEY1-KD PLC/PRF/5 cells transfected with Flag-tagged HEY1 constructs.

**G** The S40A mutant disrupted USP28/HEY1 interaction in cells. IB analysis of WCL and IP derived from HEK293T cells transfected with constructs indicated. A total of 36 h after transfection, cells were treated with 10 μM IMD-0354 for 12 h before they were harvested.

**H** Co-IP analysis of USP28/HEY1 interaction in HEK293T cells with expressing the indicated constructs.

**I** In *vivo* ubiquitination analysis of HEY1 in HEK293T cells expressing the indicated Flag-tagged HEY1 constructs, either in the presence or absence of USP28 overexpression. A total of 36 h after transfection, cells were treated with 20 μM MG132 for 8 h before they were harvested.

**J** IKKβ inhibitor IMD-0354 treatment induced HEY1 polyubiquitination. HEK293T cells transfected with indicate constructs were lysed for anti-Flag immunoprecipitation (IP) and IB.

**Supplementary Fig 7. The USP28-HEY1 axis positively regulates PD-L1 expression.**

**A** The association between CTL and overall survival (OS) of HCC patients with distinct USP28 levels.

**B** Correlation analysis between USP28 expression and immune cell infiltration (CD8+ T cells, CD4+ T cells, NK cells) using TISIDB datasets.

**C** Kaplan-Meier survival analysis of HCC patients from TCGA datasets stratified by USP28, CD4, and CD8 expression levels.

**D** USP28 expression was positively corrected with the CD274 (PD-L1) expression.

**E** IB analysis of PD-L1 protein levels in HEK293T cells expressing increasing amount of Myc-USP28. Cells were harvested 36 h after transfection with indicated plasmids.

**F** IB analysis of Pd-l1 protein levels in Hepa1-6 cells upon Usp28 depletion by shRNA knockdown.

**G** IB analysis of PD-L1 protein levels in HEK293T cells expressing HA-HEY1. Cells were harvested 36 h after transfection with indicated constructs.

**H-L** IB analysis of PD-L1 protein levels in indicated cells treated with increasing concentration of USP28 inhibitor AZ-1 for 8 h (**J**, **K**, **L**) or 25 μM for increasing amount of time (**H**, **I**) before harvesting.

**M, N** Nuclear and cytoplasmic fractions were prepared from Hepa1-6 cells upon Usp28 depletion by shRNA knockdown (**M**) or treated with 25 μM AZ-1 for 8 h (**N**). Nuclear Lamin B1 and cytoplasmic α-tubulin were used as controls.

**O, P** Cell surface Pd-l1 expression was analyzed by flow cytometry in Hepa1-6 cells following Usp28 or Hey1 depletion via shRNA knockdown (**O**), or treatment with 25 μM AZ-1 for 4 or 8 h (**P**).

**Supplementary Fig 8. USP28-HEY1 axis modulates tumor cell-intrinsic immune response to regulate anti-tumor immunity.**

**A, B** T cell-meditated tumor cell killing assay in Hey1 or Usp28 knockdown Hepa1-6 cells (**A**), or USP28 inhibitor AZ-1 treatment (**B**). Activated T cells and tumor cells were co-cultured in 24-well plates for 24 h and then surviving tumor cells were visualized by Live-Dead Cell Staining. Data are presented as mean ± SEM from three independent experiments. Data were analyzed by two-tailed unpaired *t* test, ** *P* < 0.01, *** *P* < 0.001. Scale bars, 20 μm.

**C** T cell-meditated tumor cell killing assay in USP28 inhibitor AZ-1 treatment. Activated T cells and tumor cells were co-cultured in 96-well plates for 24 h and then Caspase-3/7 activation levels were analyzed. Data are presented as mean ± SEM from three independent experiments. Data were analyzed by two-tailed unpaired *t* test, *** *P* < 0.001.

**D, E** T cell-meditated tumor cell killing assay in Hey1 KD Hepa1-6 cells treated with 25 μM AZ-1 treatment. Activated T cells and tumor cells were co-cultured in 24-well plates for 3 days and then surviving tumor cells were visualized by crystal violet staining. Relative fold ratios of surviving cell intensities are shown (**D**). Activated T cells and tumor cells were co-cultured in 96-well plates for 24 h and then Caspase-3/7 activation levels were analyzed (**E**). Data are presented as mean ± SEM from three independent experiments. Data were analyzed by two-tailed unpaired *t* test, ** *P* < 0.01, *** *P* < 0.001.

**F** Volcano plot showing differential gene expression for RNA-seq results from shUsp28 or versus NT shRNA Hepa1-6 cells.

**G** Gene-set enrichment analysis (GSEA) showing differential expression of innate immune signaling genes, cytokine-cytokine receptor interaction genes, and B cell differentiation in shUsp28 or versus NT shRNA Hepa1-6 cells. n = 3 biologically independent samples per group.

**H** Gene-set enrichment analysis (GSEA) and heatmap showing differential expression of PD-1 response genes in shUsp28 or versus NT shRNA Hepa1-6 cells. n = 3 biologically independent samples per group.

**I** mRNA levels of genes from the MHC-dependent antigen processing and presentation pathway in shUsp28 or versus NT shRNA Hepa1-6 cells (RNA-Seq results). n = 3 biologically independent samples per group.

**J** mRNA levels of indicated genes in HCC patients in TCGA were analyzed under condition of USP28 high or low. Statistical significance was assessed using the Wilcoxon test (* *P* < 0.05).

**K** Flow cytometry was used to assess H2K^d^/H2D^d^ cell surface expression in Hepa1-6 cells after 4 or 8 h of AZ-1 treatment.

**L** IB analysis was performed to assess the levels of the indicated proteins in Hepa1-6 cells treated with a USP28 inhibitor and expressing Flag-tagged HEY1 constructs.

**M** Cell surface H2K^d^/H2D^d^ on different conditions was analyzed by flow cytometry in Hepa1-6 cells.

**N, O** Hey1 depletion affects tumor growth in NOD/SCID and C57BL/6 mouse xenograft (n = 7). Hey1 depletion was achieved by shRNA in Hepa1-6 cells. Mice were sacrificed 15 days after implantation. Tumor image and tumor weight are presented (**N**). Tumor growth was measured at the indicated time points (**O**). Data are presented as mean ± SEM. *** *P* < 0.001.

**Supplementary Fig 9. USP28 expression inversely correlates with CTL infiltration and survival.**

**A** Cd8^+^ T cell density was quantified after various treatments. The quantification of GzmB and Tim-3 were represented as percentage of Cd8^+^ TILs in Hepa1-6 tumors after indicated treatments in Fig. 7d. n = 4. *t*-test. * *P* < 0.05, ** *P* < 0.01, *** *P* < 0.001,.

**B** The survival curves of USP28-low and USP28-high patient groups dichotomously divided by USP28 expression in tumors across multiple cancer types using the PanCanSurvPlot (<https://smuonco.shinyapps.io/PanCanSurvPlot/>) dataset.

**C** The association between CTL and OS in tumors across multiple cancer types with distinct USP28 expression levels within the TIDE dataset.
