# Supplementary figures and images for: IKKβ and USP28 Regulate HEY1 Stability to Promote Cancer Stemness and Immune Evasion in Hepatocellular Carcinoma
Source: Adv Sci (Weinh). 2026 May 26:e75843. Online ahead of print. doi: 10.1002/advs.75843 (PMC13335928; doi:10.1002/advs.75843)

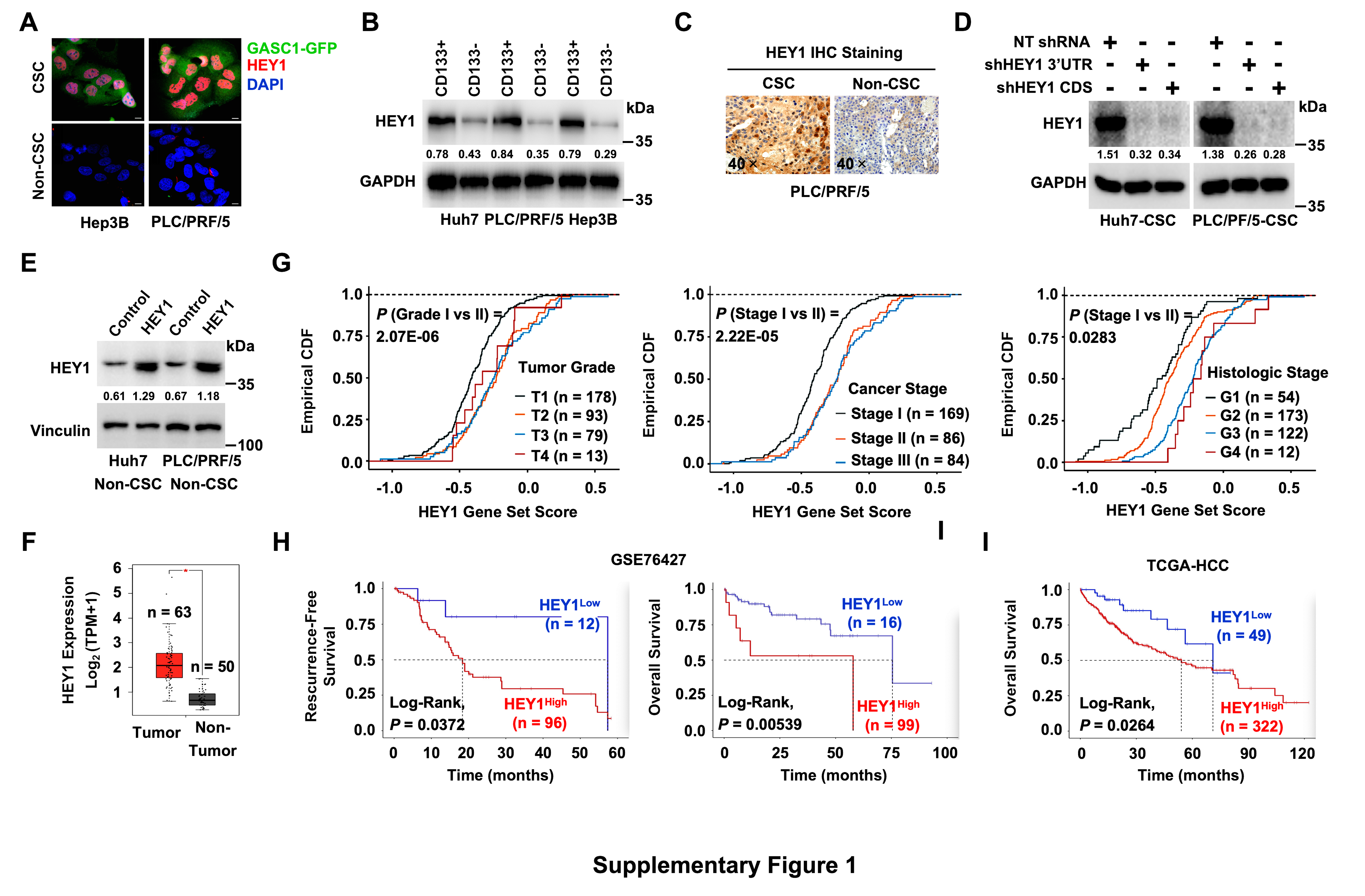

Supplement: Supplementary file 2 — Supporting File 2: advs75843‐sup‐0002‐FigureS1‐S9.zip. [file ADVS-9999-e75843-s002.zip › Supplementary Figure 1.tif]

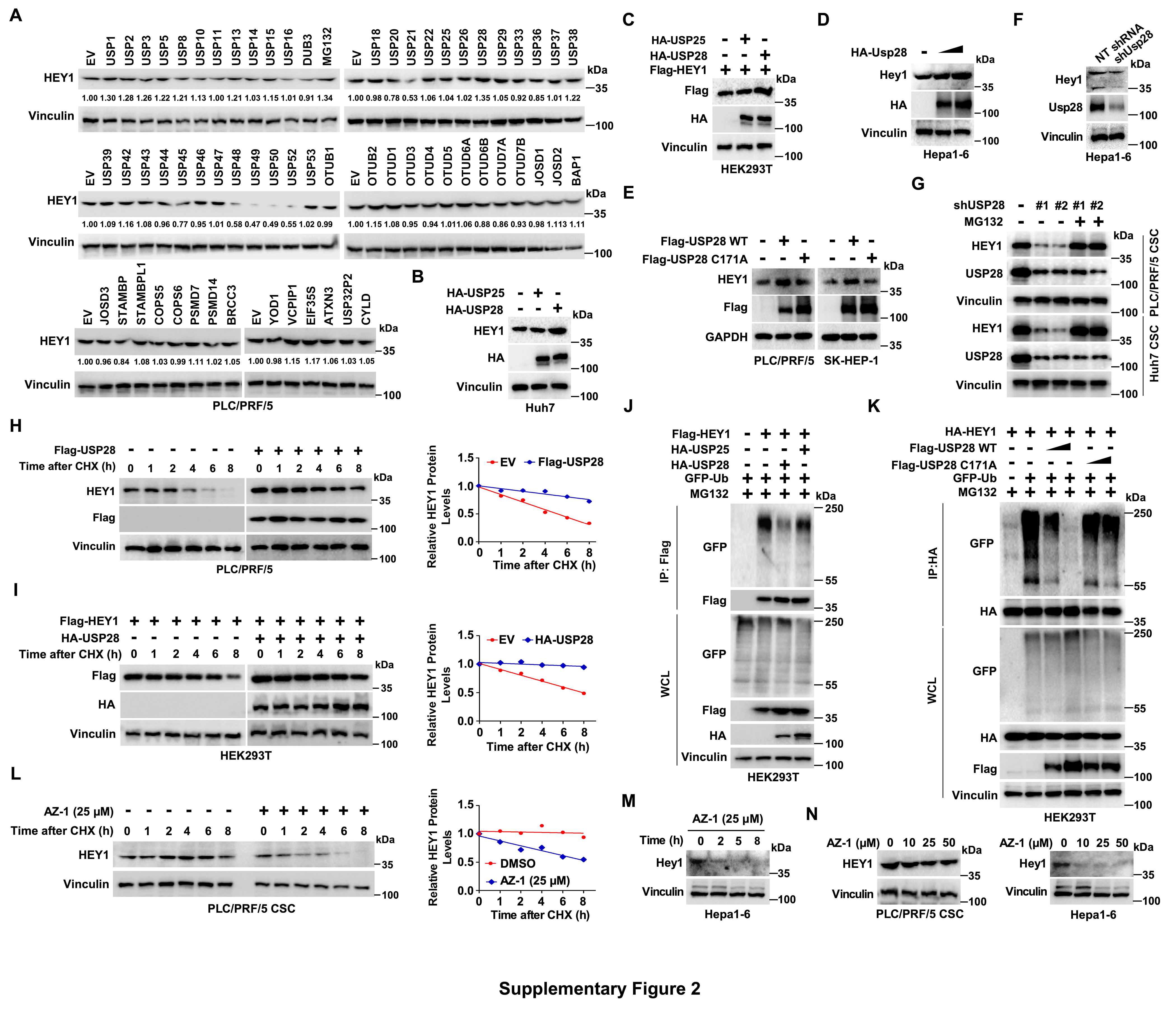

Supplement: Supplementary file 2 — Supporting File 2: advs75843‐sup‐0002‐FigureS1‐S9.zip. [file ADVS-9999-e75843-s002.zip › Supplementary Figure 2.tif]

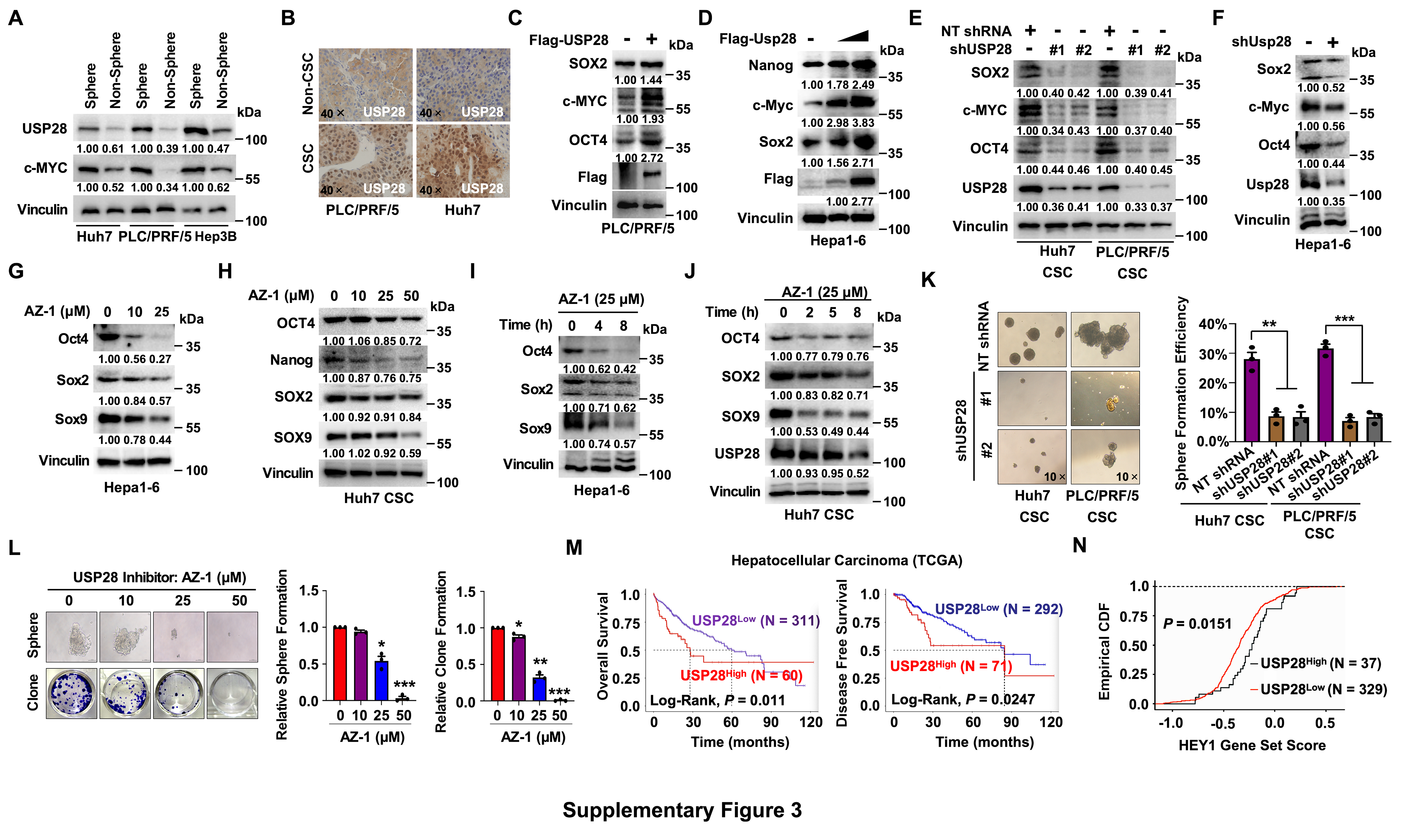

Supplement: Supplementary file 2 — Supporting File 2: advs75843‐sup‐0002‐FigureS1‐S9.zip. [file ADVS-9999-e75843-s002.zip › Supplementary Figure 3.tif]

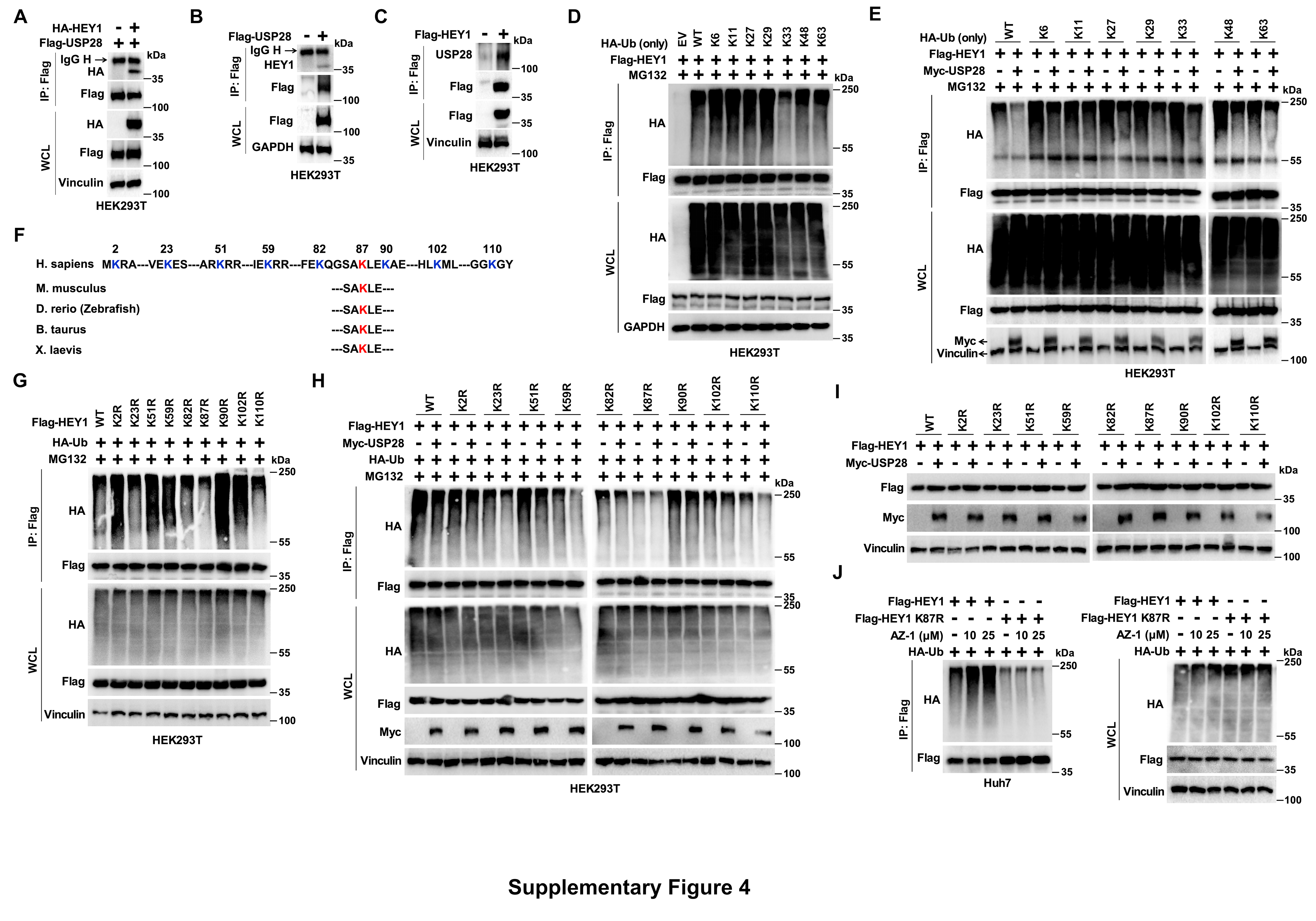

Supplement: Supplementary file 2 — Supporting File 2: advs75843‐sup‐0002‐FigureS1‐S9.zip. [file ADVS-9999-e75843-s002.zip › Supplementary Figure 4.tif]

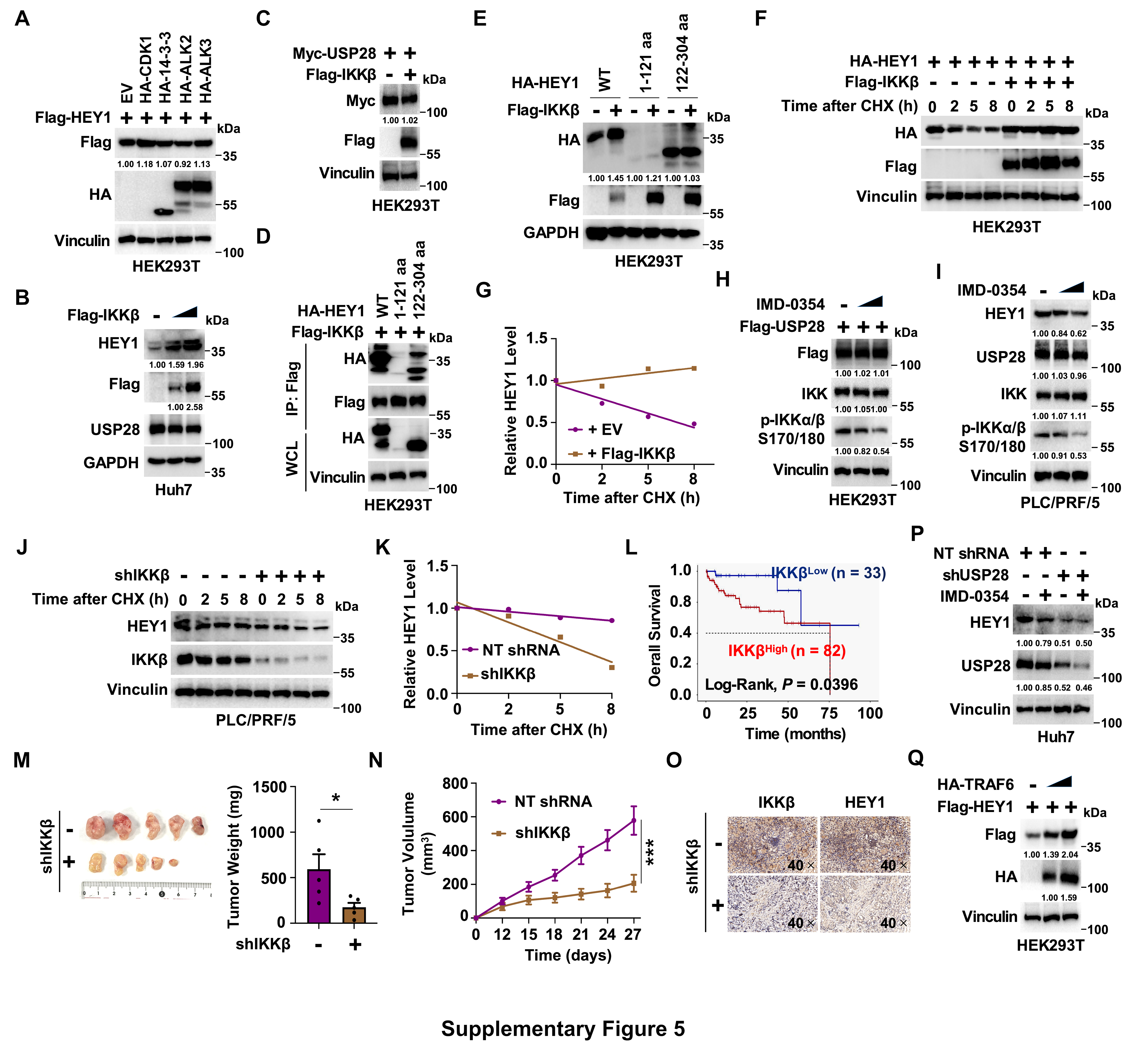

Supplement: Supplementary file 2 — Supporting File 2: advs75843‐sup‐0002‐FigureS1‐S9.zip. [file ADVS-9999-e75843-s002.zip › Supplementary Figure 5.tif]

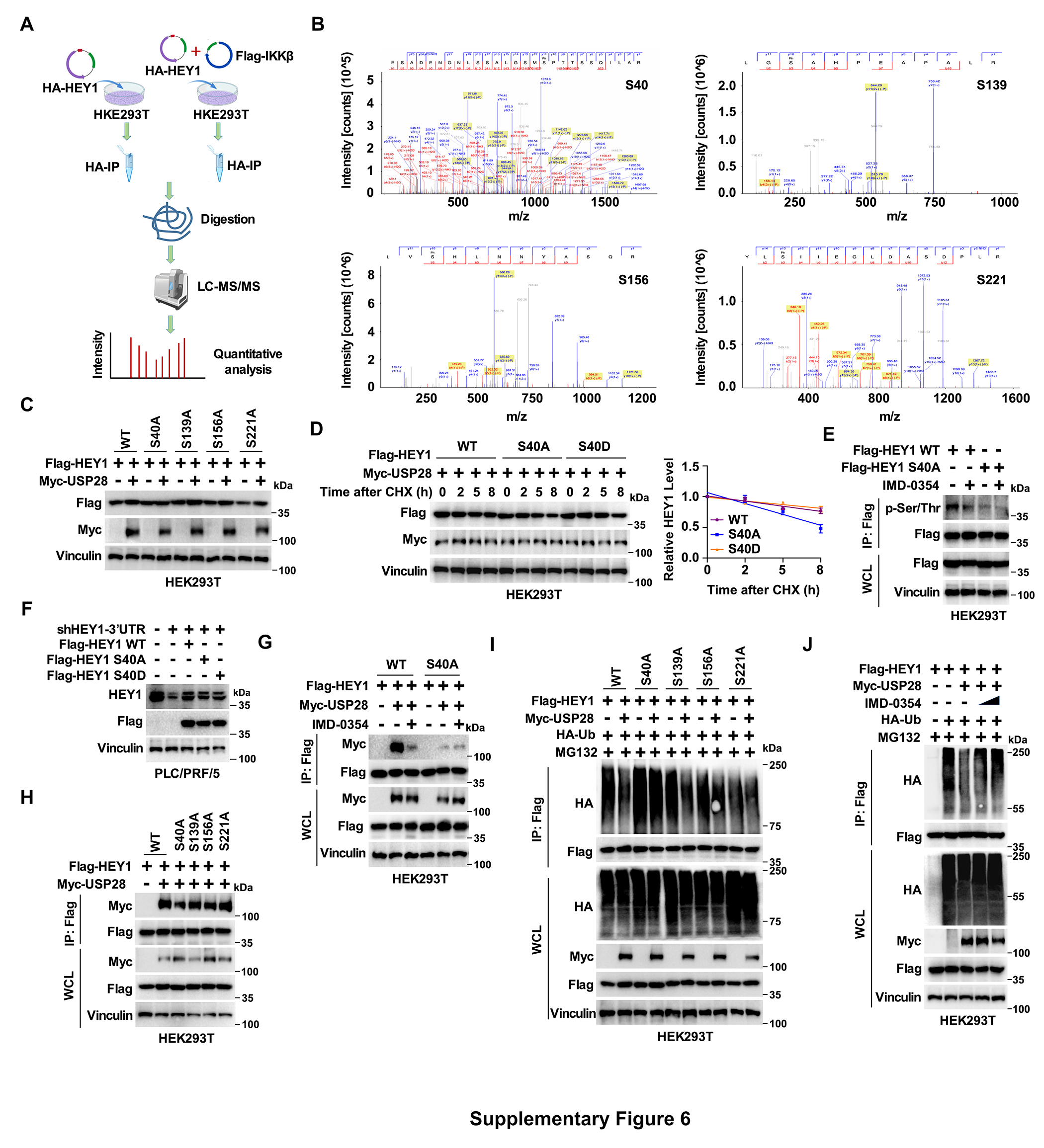

Supplement: Supplementary file 2 — Supporting File 2: advs75843‐sup‐0002‐FigureS1‐S9.zip. [file ADVS-9999-e75843-s002.zip › Supplementary Figure 6.tif]

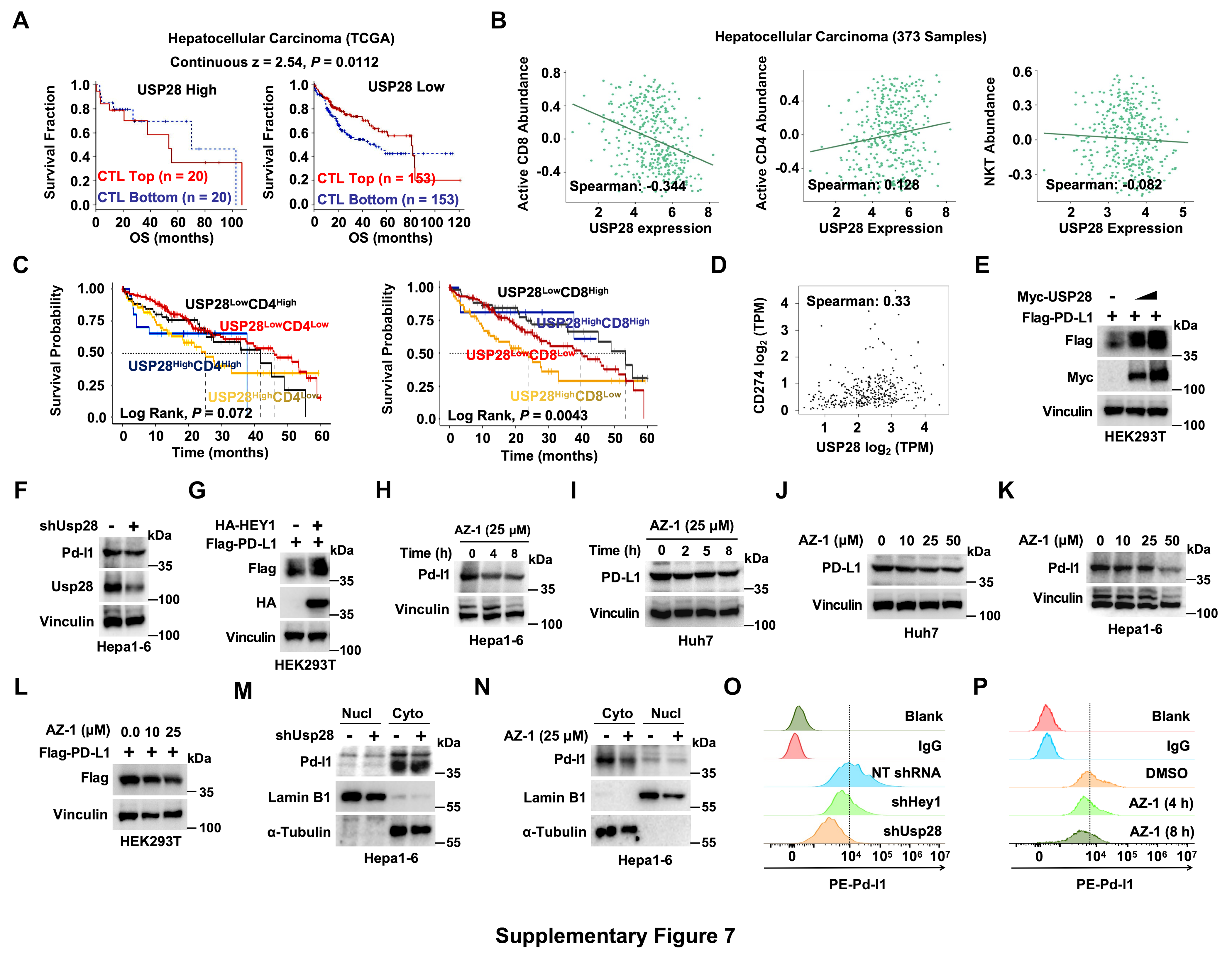

Supplement: Supplementary file 2 — Supporting File 2: advs75843‐sup‐0002‐FigureS1‐S9.zip. [file ADVS-9999-e75843-s002.zip › Supplementary Figure 7.tif]

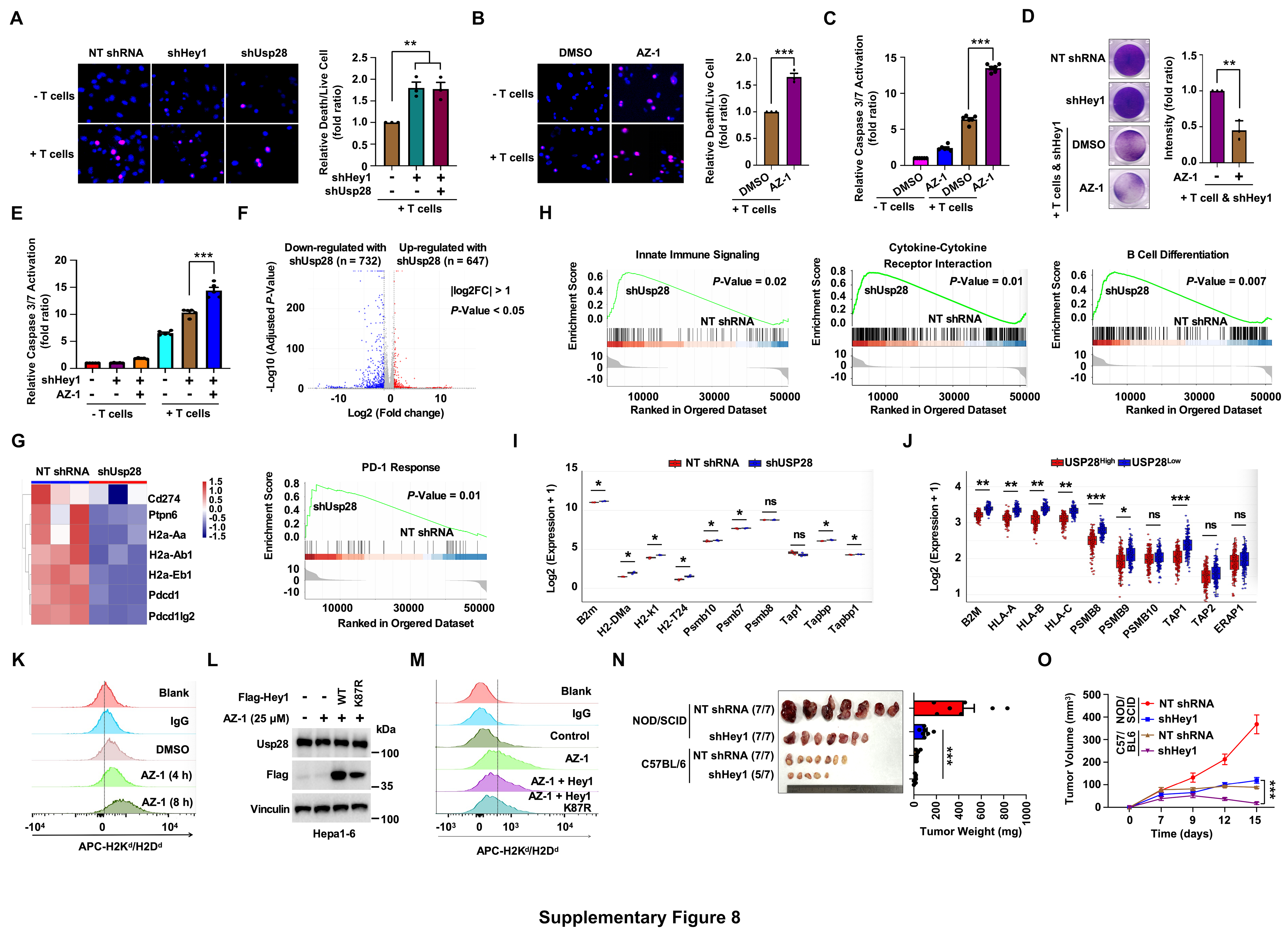

Supplement: Supplementary file 2 — Supporting File 2: advs75843‐sup‐0002‐FigureS1‐S9.zip. [file ADVS-9999-e75843-s002.zip › Supplementary Figure 8.tif]

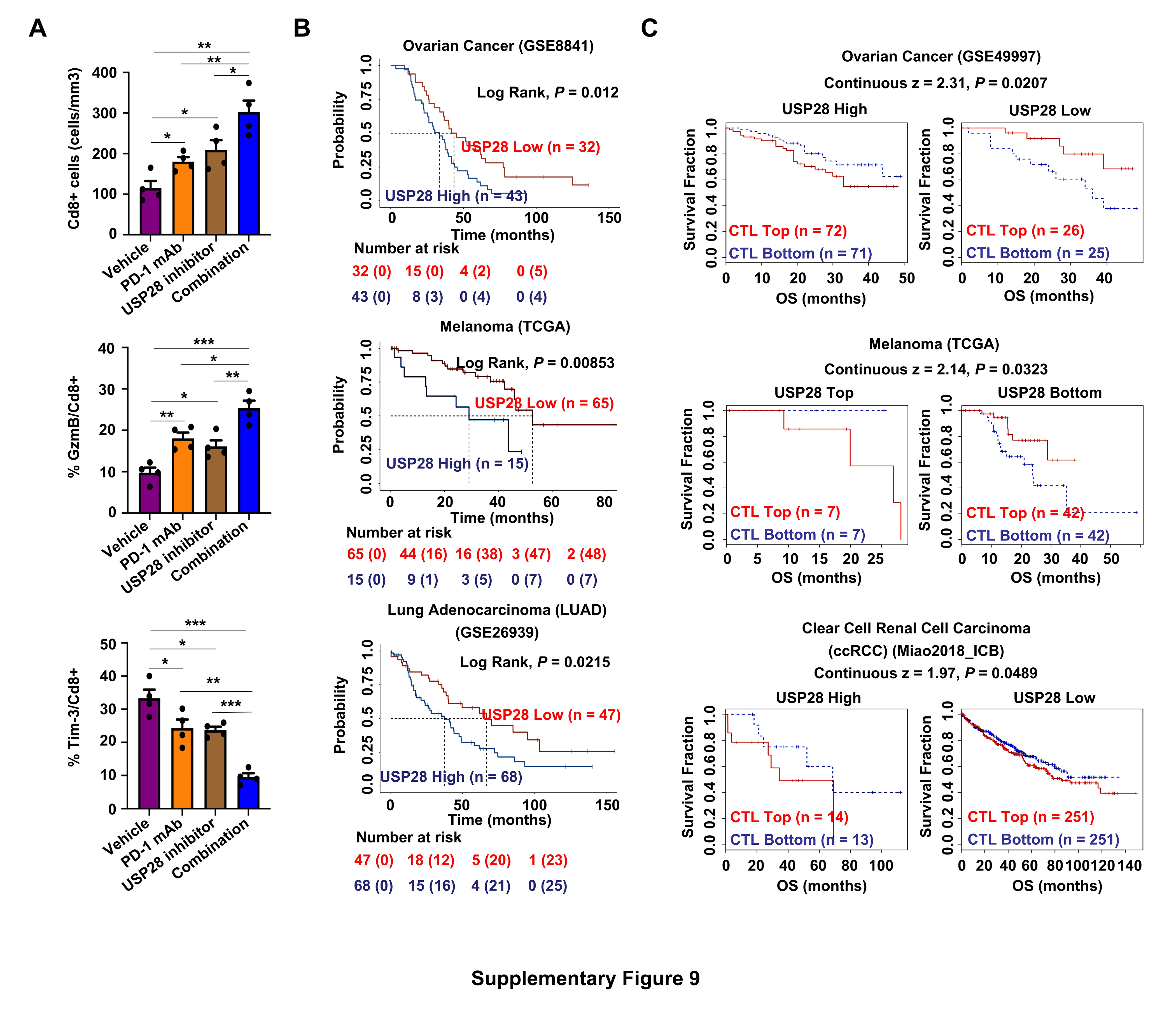

Supplement: Supplementary file 2 — Supporting File 2: advs75843‐sup‐0002‐FigureS1‐S9.zip. [file ADVS-9999-e75843-s002.zip › Supplementary Figure 9.tif]
